# Climate change effect on the widely distributed Palearctic plant bug species (Insecta: Heteroptera: Miridae)

**DOI:** 10.7717/peerj.18377

**Published:** 2024-11-22

**Authors:** Anna A. Namyatova, Polina A. Dzhelali, Veronica D. Tyts, Alexander A. Popkov

**Affiliations:** 1 Laboratory of Insects Taxonomy, Laboratory of Insects Taxonomy, Zoological Institute of Russian Academy of Sciences, St Petersburg, Russia; 2 Laboratory of Phytosanitary Diagnostics and Forecasts, Laboratory of Phytosanitary Diagnostics and Forecasts, All-Russian Institute of Plant Protection, St Petersburg, Russia

**Keywords:** Insects, Mirini, Environmental niche modeling, Global warming, Distribution, Climatic variables, Pests

## Abstract

Insects are poikilothermic organisms and temperature increase usually accelerates their development rates, population and distribution area growth. Therefore, it is assumed that global warming can be beneficial for the pests and other widespread species at least in the relatively cool temperate zones. However, climate change’s effect on the widespread species in the Palearctic remains poorly studied. This work was performed on three plant bug species (Insecta: Heteroptera: Miridae), at present inhabiting Europe and Asia. *Liocoris tripustulatus* is known from the Western Palearctic, *Lygocoris pabulinus* occupies the territories from Western Europe to South Asia, *Lygus punctatus* is distributed from Northern Europe to the Far East. In this paper, it is tested whether temperature rise is positively connected with the area of preferred climatic conditions for those species, and explores the particular climatic variables which can be limiting for the distribution of those species. Maxent software was used for the environmental niche modeling and to find the variables with significant contribution to the climatic models for the studied species. Based on those models, areas with preferred climatic conditions over different periods were calculated in QGIS. Principal component analysis and logistic regression were performed to find the variables highly contributing to the differences between the species. The results contradict the assumption that temperature growth alone can be a predictor for the widespread species and pest distribution range change. All species differ in suitable climatic conditions and their area dynamics in time, and the temperature affects each species differently. Only *Liocoris tripustulatus* might significantly expand its distribution area by 2070 due to the climate change. The areas in Asia and above the polar circle will be more suitable by that time for all three species than now. However, conditions in Europe might be less suitable for *Lygocoris pabulinus* and *Lygus punctatus* in the future. Both, temperature and precipitation variables, can be important for shaping distribution of *Liocoris tripustulatus* and *Lygocoris pabulinus*. Mean annual temperature and temperature in winter, most probably, limit the distribution of at least *Liocoris tripsutulatus* and *Lygus punctatus*, but changes in this variable affect those two species differently.

## Introduction

Understanding the dynamics of widespread species is an essential part of studying global biodiversity patterns. Such species usually represent the important component of biotopes, and changes in their distribution and population densities can lead to drastic consequences for the environment. Moreover, widespread species are often pests or potential pests, and studying their distribution dynamics is closely connected to crop protection management (*e.g.*, Lehmann et al., 2020). Many ecological factors influence species distribution, and it is usually impossible to account for all of them simultaneously. Studying separate variables helps answer the complex question of how species respond to environmental changes.

Climate is an important factor in determining species distribution (*e.g.*, Prakash et al., 2014; Skendžić et al., 2021). It is usually assumed that the rise in temperature leads to population density increase and geographic range growth in pests and other widely distributed species (Bale et al., 2002; Dukes et al., 2009; Deutsch et al., 2018; Skendžić et al., 2021). This means that the areas of preferred climatic conditions of such species should show direct dependency on temperature changes.

The presence of such trends has been insufficiently tested. Although numerous papers have aimed at finding the preferable conditions using environment niche modeling (ENM) for pests and projecting them to other periods, they mostly address finding possible distribution changes during the past or forecasting them for the future (*e.g.*, Gilioli et al., 2014; Barredo et al., 2015; Dellicour et al., 2017; McAvoy et al., 2017; Song et al., 2018; Avtaeva et al., 2019; Gao et al. 2022). The last glacial maximum (LGM) was 3–5 °C cooler than today (Meehl et al., 2007). It is widely accepted that during this period, many temperate species, including those with current wide distribution, persisted in the southern refugia (*e.g.*, Wahlberg & Saccheri, 2007; Stewart et al., 2010; Habel et al., 2010; Eberle et al., 2021). However, whether global cooling led to range contractions or range shifts was not tested for many widespread species. Even if range contraction occurred, this might not necessarily be a direct consequence of temperature decrease alone. At least partly, the distribution ranges might be affected by the ice shield covering most of Europe, which reduced the food supply and destroyed the biotopes (*e.g.*, Hewitt, 1999; Clark & Mix, 2002). The studies addressing possible species distribution changes in the future demonstrated that widespread species and pests can react to climate warming differently and that the abundance and distribution areas of certain species might decrease, leading to these species potentially facing extinction (Stiling & Cornelissen, 2007; Shrestha, 2019; Lehmann et al., 2020). Although climate scenarios predict a global temperature rise in the future, distribution ranges can be affected by other factors or complex environmental interactions (*e.g.*, Hoover & Newman, 2004).

If global temperature fluctuations alone can be a predictor of widespread species distribution changes, areas of preferred climatic conditions dynamics should reflect temperature dynamics in different periods. In the last interglacial period (LIG), the temperature was higher than in the Holocene. At least in the Palearctic, the temperature in the middle of the Holocene (MH) was higher than it is today, and it is predicted that during the 21st century, the temperature will be higher than it is today and, most probably, will exceed that during the LIG (Van Vuurenb et al., 2008; Field et al., 2014; Kaufman et al., 2020; Bova et al., 2021; Essell et al., 2023). Therefore, the areas of preferred climatic conditions of widely distributed species in the MH should be narrower than during the LIG but somewhat wider than that today, and they should considerably expand in the future, approaching or even exceeding those areas during the LIG.

We selected three species known from Europe and Asia with different recorded distributions. We chose these species as they can be positively identified morphologically or using molecular data. *Lygocoris pabulinus* (Von Linné, 1761) adults live in meadows and can be univoltine or multivoltine, with their first-generation larvae developing on trees (Saulich & Musolin, 2020). This trans-Palearctic species ranges from Western Europe to East Asia. It is also widely distributed in North America (Kerzhner & Josifov, 1999). *Liocoris tripustulatus* (Fabricius, 1781) inhabits wide areas from Western Europe to the Ural Mountains and is also known from Central Asia and Iran (Kerzhner & Josifov, 1999; Kozminykh, 2019; Esenbekova & Orynbek, 2021; Çerçi & Özgen, 2021). Its main host plant is *Urtica dioica* L. *Lygus punctatus* (Zetterstedt, 1840) is known from the Western Palearctic to the Russian Far East; it is also recorded from Kazakhstan (Aglyamzyanov, 1990; Kerzhner & Josifov, 1999). All three species are known as pests (*e.g.*, Kytö, 1992; Wheeler, 2001; Groot et al., 2003; Wynde & Port, 2012; Steenman et al., 2023).

It was previously shown that widespread Palearctic species with different distribution types have different environmental preferences (Namyatova, 2020; Namyatova, 2022). One can test which particular variables contribute to the species distribution differences using principal component analysis (PCA) and logistic regression. Although there are studies applying PCA for these purposes (*e.g.*, Wellenreuther, Larson & Svensson, 2012; Ačanski et al., 2017), we could not find any similar works addressing the species distribution differences using logistic regression.

This study has two main aims. First, we test whether the dynamics of the areas of preferred climatic conditions for three Palearctic widespread species are similar and correspond to the global temperature fluctuations in the Pleistocene starting from the LIG. The second aim is to find and compare the climatic variables that contribute to the species niche models and geographic range differences the most and, therefore, can limit their distribution.

## Material and Methods

**Species identification.** The study was based on the collection preserved at the Zoological Institute of the Russian Academy of Sciences (ZISP), hosting ca. 700 specimens of *Liocoris tripustulatus*, ca. 1,000 specimens of *Lygocoris pabulinus*, and ca. 320 specimens of *Lygus punctatus*. The collection event data was entered into the Arthropod Easy Capture Database (https://research.amnh.org/pbi/locality/index.php). The external view of specimens from all series was checked for correct species identification. *Liocoris* is a monotypic genus, and no species can be confused with *Liocoris tripustulatus*. Nevertheless, we examined the male and female genitalia of at least 10 specimens of each sex to confirm their consistency. *Lygus punctatus* can be identified by the combination of punctation on the hemelytron, color, and male genitalia (Namyatova, Tyts & Bolshakova, 2023). Therefore, the male genitalia of the specimens from most of the series were dissected. We included only the specimens that were positively identified. The shape of the right paramere is important for the identification of *Lygocoris pabulinus* (Kerzhner & Jaczewski, 1964; Yasunaga, 1991; Lu & Zheng, 2001). This feature was examined in all the series of this species.

**Dissections.** The genital capsule or entire abdomen was removed and heated in 10% KOH for 2–5 min. Subsequently, these structures were washed in clean water and dissected in glycerol.

**Localities selection for ENM**. We took the localities from four sources. First, we used the ZISP collection, which is the largest collection of the Palearctic Miridae (Konstantinov & Namyatova, 2019). Second, the localities from the Genbank entries were added if the sequences corresponded to the assigned species. Third, the localities from iNaturalist were used if we could positively confirm their identification using the digital images provided with the record. Fourth, data from previously published papers were added for *Liocoris tripustulatus* and *Lygocoris pabulinus* but not for *Lygus punctatus* because this species can be easily misidentified even by experienced taxonomists. Overall, our dataset comprised 131 localities for *Lygus punctatus*, 497 for *Liocoris tripustulatus*, and 401 for *Lygocoris pabulinus* (Data SI1).

**Spatial filtering**. The data in this study is biased because more collecting efforts were undertaken in the Western Palearctic. Additionally, most of the specimens were collected along major roads and easily accessed areas. This bias can be addressed by reducing the number of occurrence records in oversampled regions using spatial filtering. For *Liocoris tripustulatus*, we used GeographicDistanceMatrixGenerator ver. 1.2.3 (Ersts, 2023) to create the matrix with the distances between each pair of occurrence records. Only the records removed from each other at distances >50 km were left. Preference was given to more recent occurrences. For two other species, a script in R to perform the same function automatically was created (the script is available at https://github.com/vtyts/ThreeMirini, DOI 10.5281/zenodo.13348219). Overall, 246 occurrence records were left for *Lygocoris pabulinus*, 243 for *Liocoris tripustulatus*, and 105 for *Lygus punctatus*. These datasets were used for the PCA, logistic regression, and Maxent analysis.

**Maps**. All the layers have 5-arc min (∼10 km) resolutions. This resolution was chosen as a trade-off between computational efficiency and climate data accuracy (*e.g.*, Araújo et al., 2005; Seo et al., 2009). Additionally, the coordinates for many localities in our dataset are approximate, and higher resolution might lead to erroneous interpretations (Graham et al., 2004; Hanberry, 2013). Nineteen bioclimatic variables were used (O’Donnell & Ignizio, 2012). The layers for the LIG (115,000–130,000 years ago) were downloaded from the Paleoclim website (http://www.paleoclim.org). Other layers were downloaded from Worldclim v.1.4 ( https://www.worldclim.org/data/v1.4/worldclim14.html): current conditions (the years 1960–1990), the LGM (19–29 Kya), the MH (5–7 Kya), 2070 with the lowest CO_2_ emissions (rcp26), and the highest CO_2_ emission (rcp85) scenarios. Worldclim layers, created with three climate models, *i.e.*, MPI-ESM (MPI-ESM-P for the past conditions, and MPI-ESM-LR for the future conditions), MIROC-ESM, and CCSM4, were downloaded. These three models were chosen because they are significantly different from one another (Knutti, Masson & Gettelman, 2013), and all of them provide layers for the LGM, the MH, and future conditions. The study aims to find and compare the climatic variables contributing the most to the species’ distribution, therefore, all 19 variables were used as an initial baseline for the niche modeling, PCA and linear model analyses.

All the layers were converted to ASCII format using QGIS ver. 3.32 and trimmed to the Palearctic (20°N–90°N, −30°W–180°E) using DivaGIS. “Sample with data” files (swd) were created either in QGIS ver. 3.32 or using the *raster* package in R (Hijmans et al., 2023).

**ENM and projections.** The Maxent software (version 3.4.1) (Phillips, Anderson & Schapire, 2006) was chosen because it performs well with the presence-only datasets and can create robust results for the irregularly sampled data. It is applicable to our datasets with occurrences taken from different sources (Elith et al., 2006; Elith et al., 2011; Pearson et al., 2007; Kramer-Schadt et al., 2013). The models were built using swd files and bioclim layers in ASCII format. Ten replicates of Bootstrap run type with 25% of localities assigned for the random test percentage were applied. Using a bias file for the background data is an additional way to address the biased dataset and to increase the performance of the model (Kramer-Schadt et al., 2013). A bias file based on the coordinates of the occurrence points was created as a two-dimensional kernel density estimate using the kde2d function from the *MASS* package (Elith, Kearney & Phillips, 2010; Fourcade et al., 2014; Ripley et al., 2020) in R. Projections for the past and future conditions were performed using the Maxent software. A bias file was used in Maxent to choose 10,000 background points. The variables having percent contribution (PC) or permutation importance (PI) >10% were treated as highly contributing to the models.

**Variable selection and parameter adjustment for ENM.** Some Worldclim variables highly correlate with each other and, this can affect the model performance. There are two approaches in reducing the number of variables. First, they are pre-selected based on the deep knowledge on the biology and other characteristics of the studied taxa. Second approach is automatic removal of the least contributing variables (*e.g.*, Zeng, Low & Yeo, 2016). The second approach was chosen, because the biology is poorly known for all three species. The highly correlated variables were excluded using the *MaxentVariableSelection* package in R (Jueterbock et al., 2016; Jueterbock, 2018). It chooses the best set of variables with the lowest small-sample corrected Akaike information criterion (AICc) value based on the regularization multiplier and features. The Pearson correlation (PCor) for the variables for each species was calculated using R core functions and provided in Data SI2. Feature classes and a regularization multiplier (beta-multiplier) were tuned to avoid overfitting and over-complexity (Morales, Fernández & Baca-González, 2017).

The *MaxentVariableSelection* package was used to choose the best regularization multiplier for each feature class using AICc values. The analyses were run for the multipliers ranging from 1 to 6 and with the following feature classes combinations, *i.e.,* L, LQ, LQH, H, LQHP, and LQHPT (L = linear, Q = quadratic, H = hinge, P = parameter, T = threshold). Maxent analysis was run for all feature classes, and the best model after evaluation was selected for further analysis (see below).

**ENM evaluation.** First, the area under the curve (AUC) value was used because it is valid for model comparison over the same study area (Bohl, Kass & Anderson, 2019). The partial receiver operating characteristic (ROC) test using the NicheToolBox (https://github.com/luismurao/nichetoolbox) was applied for the evaluation of model performance as it helps avoid some problems connected with standard AUC values (Peterson, Papeş & Soberón, 2008). It was previously demonstrated that models with high differences between AUC test and training values and high omission rates (>0.1) are likely to be overfitted (Bohl, Kass & Anderson, 2019). The differences between AUC training and test values were calculated, and maximum training sensitivity plus specificity test omission values were extracted from the MaxentResult.csv files. The model with relatively high AUC values, low differences between training and test AUC, and low omission error rates was chosen for the visualization and niche comparisons. The model with the lower AUC values can be more accurate for the widespread species (*e.g.*, Lobo, Jiménez-Valverde & Real, 2005). Therefore, low omission rates were prioritized in the model evaluation. All values for the model evaluation parameters are provided in Table SI1.

**Environment niche projection areas and climatic variable ranges**. To find the areas of preferred climatic conditions, ENM projection layers were thresholded using the “maximum training sensitivity plus specificity Cloglog threshold” as the thresholds maximizing sensitivity and specificity perform well on presence-only datasets (Liu, Newell & White, 2016). The values of this threshold were extracted from the maxentResults.csv file for each model (the script is provided online: https://github.com/vtyts/ThreeMirini, DOI 10.5281/zenodo.13348219). The averaged geographic projection layers resulting from the Maxent analysis were cut using the thresholds in QGis ver. 3.32. Their area was calculated in the same software using the RasterCalculator function. To calculate the areas for Europe and Asia, the thresholded geographic projection layers were cut with the Ural mountain longitude (60°E), which is considered a formal border between Europe and Asia. For the calculation of the areas with preferable conditions in the polar regions, the geographic projections were cut with the Arctic Circle latitude (66.562°N).

The thresholded averaged geographic projections were converted into shapefiles in QGis ver. 3.32. The variable range values were extracted by trimming the bioclimatic variable layers with these shapefiles. Minimum and maximum climatic variables were extracted from the trimmed bioclimatic layers using the R script provided in the GitHub repository (https://github.com/vtyts/ThreeMirini, DOI 10.5281/zenodo.13348219).

**Maps.** All maps were generated using Maxent ver. 3.4.1 and processed in QGis ver. 3.32.

**PCA.** This analysis was run on the full variable dataset for all species to test whether we can differentiate species based on the climatic data and identify the climatic variables most responsible for the observed difference. To further analyze the level of dissimilarity among the species and ascertain the uniqueness of these variables for each species pair, we also performed a separate PCA for the reduced datasets on three species pairs (*Liocoris tripustulatus*—*Lygocoris pabulinus*, *Liocoris tripustulatus*—*Lygus punctatus*, *Lygocoris pabulinus*—*Lygus punctatus*).

All the analyses and visualizations were performed using the core functions and additional packages in R. For plot visualization, we used the following packages: *ggplot2* (Wickham, 2016), *ggrepel* (Slowikowski, 2021), *wesanderson* (Ram et al., 2023) and *scales* (Wickham & Seidel, 2020). The broken-stick model (MacArthur, 1957; Frontier, 1976) was used to determine the number of principal components (PCs) to keep (the script for this analysis is available at https://github.com/vtyts/ThreeMirini, DOI 10.5281/zenodo.13348219).

To further interpret the results of the PCA and estimate whether there was a significant difference between at least two species, an analysis of variance (ANOVA) for each statistically significant PC for each dataset was performed utilizing the lm and ANOVA functions from the *stats* package. The mean values for each significant PC for the full dataset with all three species were compared using Tukey’s test *via* the TukeyHSD function from the *stats* package to determine the pairs found to be significantly different. We consider the pair of species with a *p*-value < 0.001 as significantly different. The point range plots with mean values and confidence intervals were used for the visualization of the differences between the species. To estimate the importance of climatic variables, the loadings for each measurement for each significant PC were extracted.

**Linear model.** The logistic regression was chosen as a linear classification model to test whether climatic variables can be used for species differentiation and to find which variables can limit the distribution of each species in comparison to other studied species. In addition, we analyzed the fitted model coefficients for these features. Our modeling consisted of the following steps: feature selection, model fitting, model evaluation, and model explanation. All the analyses and visualizations were performed using the core functions and additional packages in Python 3.11: *NumPy* (Harris et al., 2020), *Matplotlib* (Hunter, 2007), *statsmodels* (Seabold & Perktold, 2010), *shap* (Lundberg & Lee, 2017), and *scikit-learn* (Pedregosa, Varoquaux & Gramfort, 2011) (the code is provided at https://github.com/vtyts/ThreeMirini, DOI 10.5281/zenodo.13348219).

The analysis was run for three subsets corresponding to each species pair. Each subset was further split into train and test subsets, which included 80% and 20% of observations, respectively. All the features were scaled with standard scaling.

All 19 variables were used for the logistic regression to test whether the differences in species distribution depend on temperature only or other variables also can be important. To mitigate the problem of multicollinearity that distorts the results of the logistic regression, all possible combinations of variables varying in size from 1 to 19 were calculated. Following this, the condition numbers were evaluated. The best combination of variables for each size of the set with the lowest condition number result was picked. To choose the size of the final set, the variation inflation factor (VIF) for each variable was calculated. The combination without VIF exceeding 10 and with the highest number of variables was selected.

L2 penalty was added to prevent overfitting. To define the coefficient for L2 penalty for each model, cross-validation with five folds on train data and the ROC–AUC metric was used. The search space of L2 penalty coefficients was defined as a grid of 100 values from 0.001 to 0.5. The final fitted models on our test subset using ROC–AUC, precision, recall, and F1-score metrics were evaluated. Regression coefficients, beeswarm plots, and partial dependence plots from the SHAP library (Lundberg & Lee, 2017) for model explanation and analysis were used.

## Results

**Figure 1:**
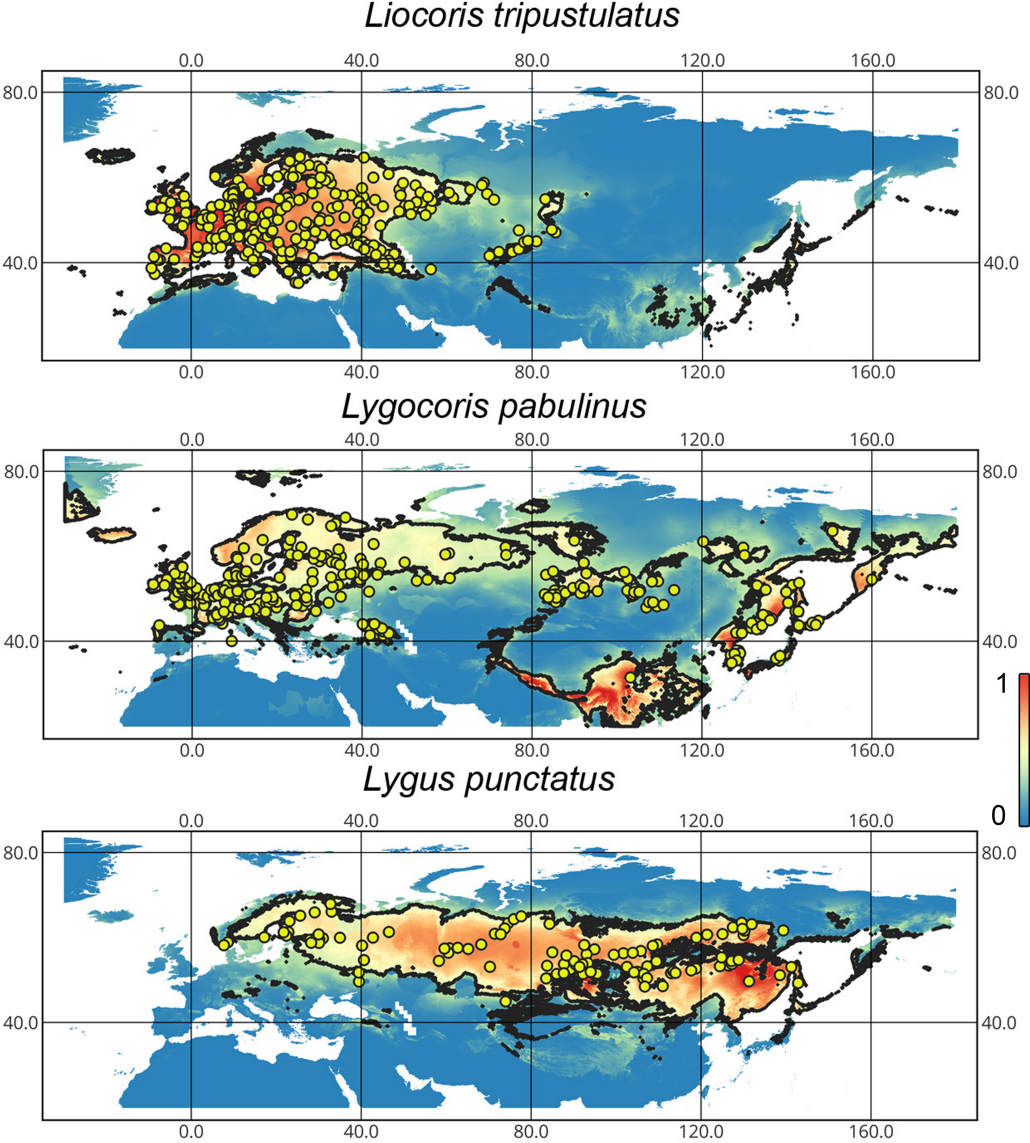
The geographic projection of the Maxent models to the present conditions. The solid line corresponds to the maximum training sensitivity plus specificity Cloglog threshold in Maxent. Colors correspond to the suitability score with dark blue (0) representing the most unsuitable places and red (1) representing the most suitable places. The yellow circles correspond to the localities where the species were collected. The figures were generated using Maxent ver. 3.4.1 (released under MIT license) and processed in QGis ver. 3.32 (licensed under Creative Commons Attribution-ShareAlike 3.0 license (CC BY-SA).

**Geographic distribution of the studied taxa.** Our results support that *Lygocoris pabulinus* has trans-Palearctic distribution. Although it was recorded from Southern Europe, the records of this species from this area are very scarce. We could not confirm the presence of this species in Central Asia, except for Mongolia. We also confirm that *Liocoris tripustulatus* mostly inhabits the Western Palearctic, and in this paper, we record this species for the first time from Western Siberia. *Lygus punctatus* is known from Western Europe to East Asia. Although it was recorded in several European countries (Kerzhner & Josifov, 1999), we could only confirm its presence in Scandinavia (Fig. 1).

**Ecological niche model performance.** The Maxent models have high discriminative power for the training and test datasets with high AUC. The training and test AUC for the *Liocoris tripustulatus* models are 0.85–0.92. For *Lygus punctatus*, they are 0.7–0.92, and for *Lygocoris pabulinus*, they are 0.74–0.89. In all the cases, the model based on the linear feature class (L) only shows the lowest AUC. The omission rate ranges are 0.08–0.19 for *Liocoris tripustulatus*, 0.16–0.19 for *Lygocoris pabulinus*, and 0.08–0.17 for *Lygus punctatus*. Partial ROC values are very similar to AUC values. All the values and the models chosen for the subsequent analyses are provided in Table SI1.

**Figure 2:**
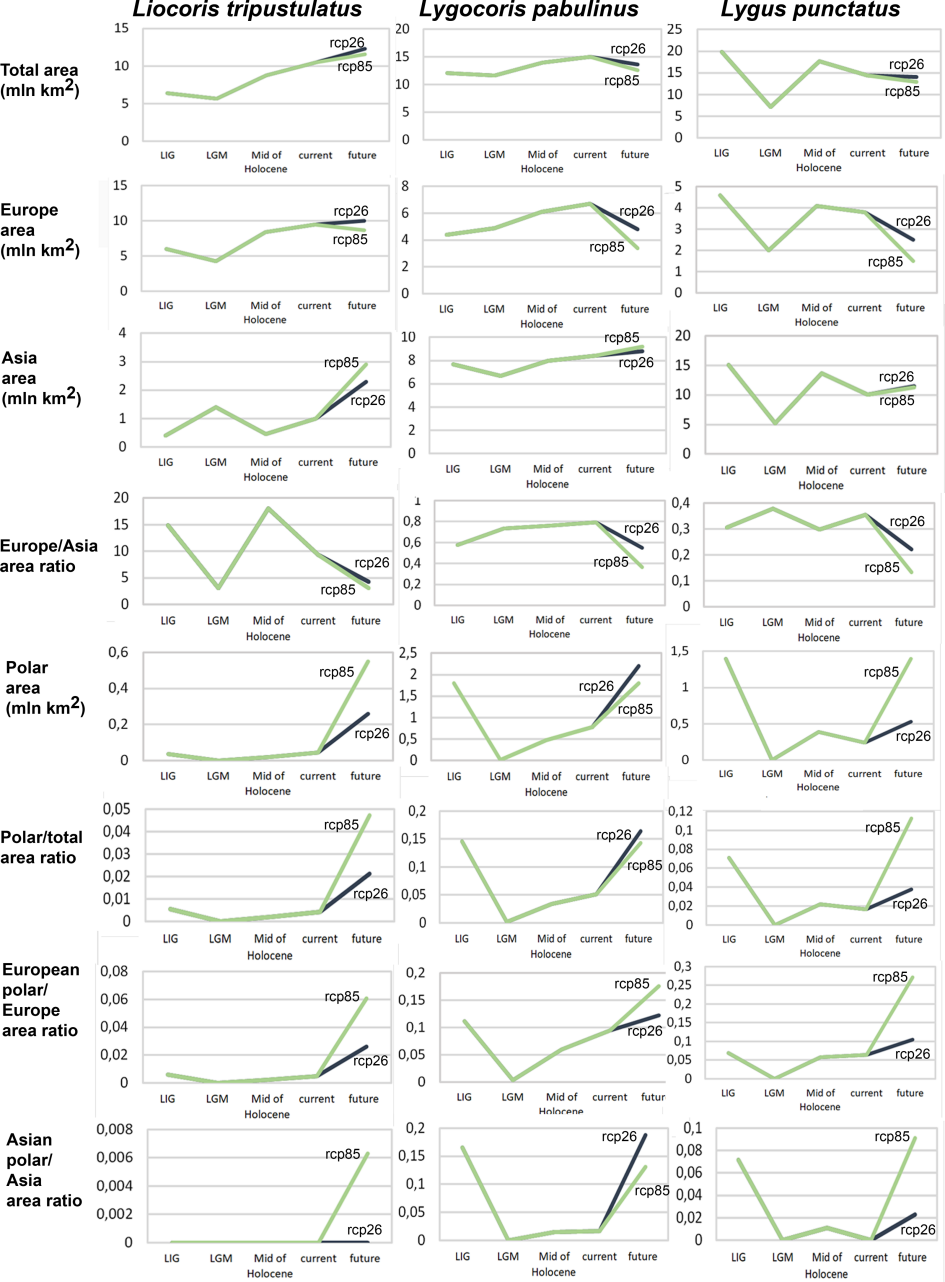
Graphs showing the areas of preferred climatic conditions dynamics in time. X-axis correspond to the different time periods. *Y*-axis correspond to the area of preferred conditions, based on the Maxent models, calculated with CR dataset and MPI-ESM climate model. For the future, green line corresponds to the rcp85 scenario (the highest CO_2_ emission), and dark blue line corresponds to the rcp26 scenario (the lowest CO_2_ emission).

**Geographic projection area dynamics in time.** The total preferable condition areas for all models, scenarios, and periods are provided in Data SI3, and the area dynamics for each species are shown in Fig. 2, Figs. SI1, SI2. Different climate models—MPI-ESM, MIROC-ESM, and CCSM4—show similar areas of preferred climatic conditions area dynamics. However, the MIROC-ESM results differ more noticeably from those obtained based on the other two models. The dynamics of the areas of preferred climatic conditions for each species did not correspond to the temperature dynamics in the Pleistocene.

**Figure 3:**
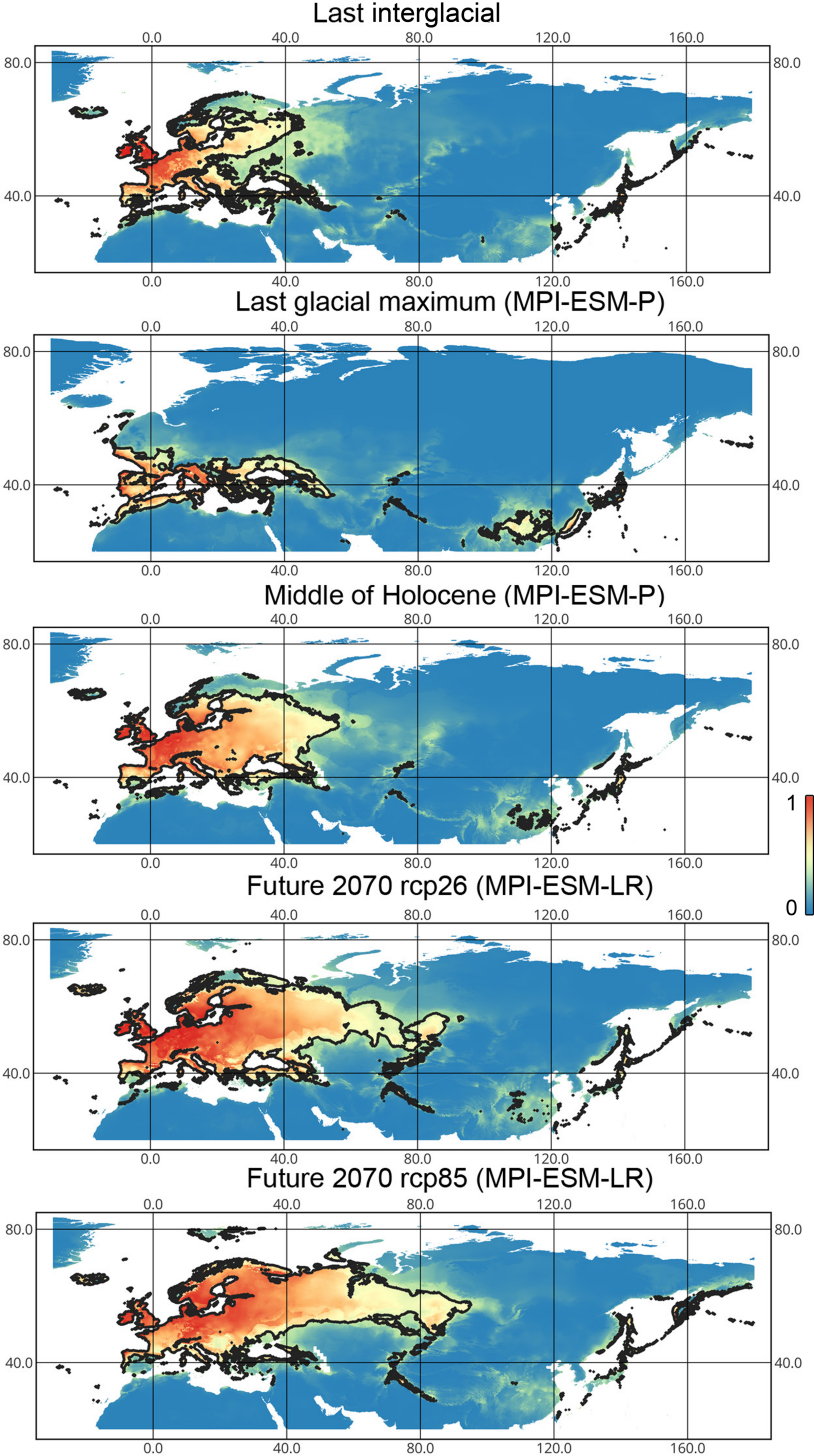
Geographic projections of the Maxent models to the past and future conditions using the MPI-ESM climate model for *Liocoris tripustulatus*. The solid line corresponds to the maximum training sensitivity plus specificity Cloglog threshold in Maxent. Colors correspond to the suitability score with dark blue (0) representing the most unsuitable places and red (1) representing the most suitable places. The figures were generated using Maxent ver. 3.4.1. (released under MIT license) and processed in QGis ver. 3.32 (licensed under Creative Commons Attribution-ShareAlike 3.0 license (CC BY-SA)).

*Liocoris tripustulatus*. The preferred climatic conditions for this species during the LIG were mostly in Europe, below the polar circle (∼6 mln km^2^). During the LGM, the refugia were probably in Southern Europe, Northern Africa, the Crimea, and the Caucasus. The preferred conditions were also in Asia, but it is unlikely that this species lived there. The total area of the preferred conditions in Asia for this period is 3–6 mln km^2^. By the MH, the preferred conditions covered most of Europe except for its northernmost regions and spread more toward Asia rather than in the LIG (∼9–10 mln km^2^). The area of the preferred conditions is slightly wider in the current period (∼10.5 mln km^2^) and reaches Western Siberia, but the conditions above the polar circle are still unsuitable for this species. *Liocoris tripustulatus* is predicted to be more widespread in Asia and polar areas in the future. The projections on the future conditions modeled under different CO_2_ emission scenarios (rcp26 and rcp85) showed that the rate of the polar area colonization might depend on the CO_2_ emission.rate (rpm26: 0.15–0.45 mln km^2^, rpm85: 0.55–1 mln km^2^). However, the total areas of the preferred climatic conditions are similar in both scenarios (rpm26: ∼11–12 mln km^2^, rpm85: 12–13 mln km^2^) (Figs. 1 and 3, Figs. SI1–SI4).

**Figure 4:**
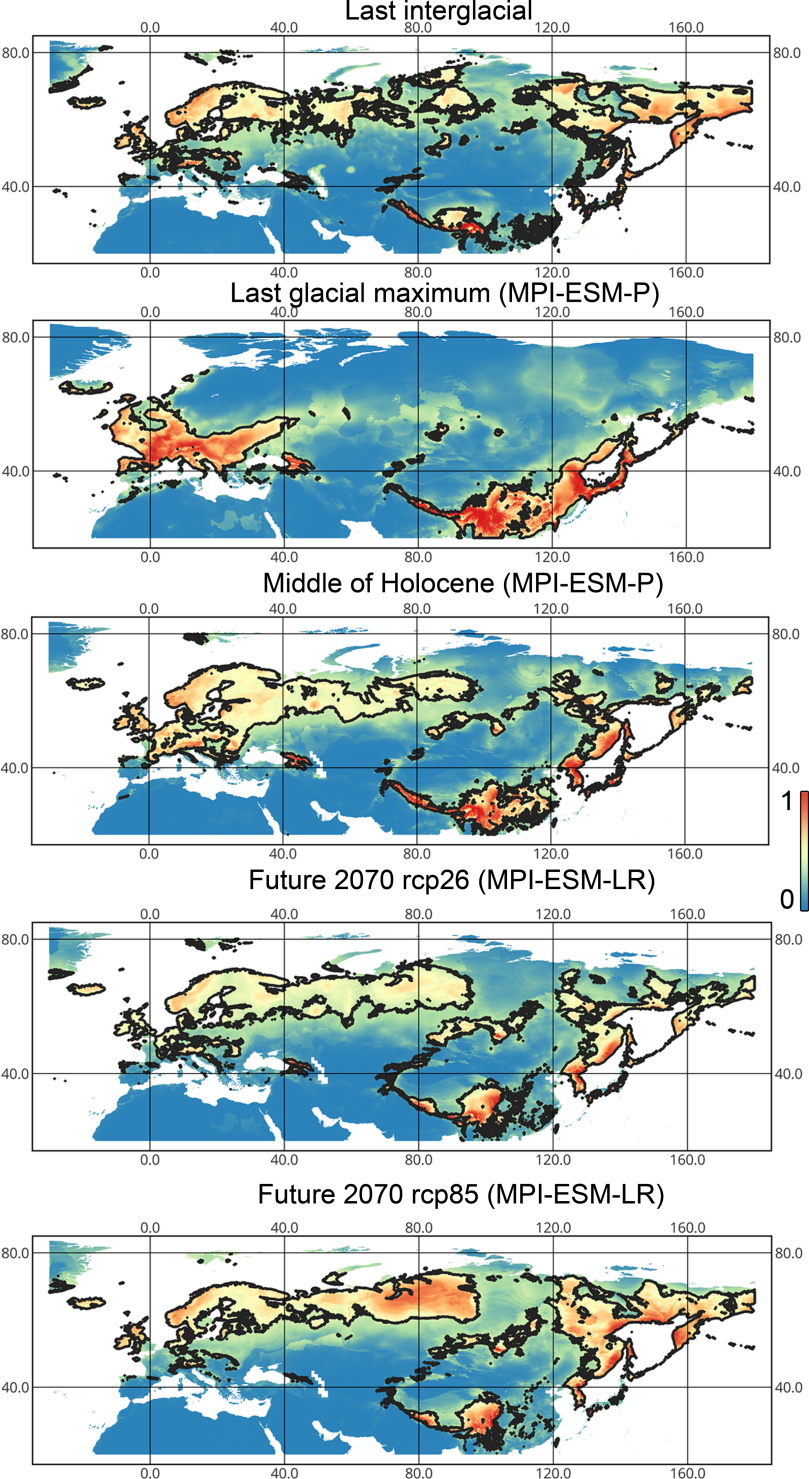
Geographic projections of the Maxent models to the past and future conditions using the MPI-ESM climate model for *Lygocoris pabulinus*. The solid line corresponds to the maximum training sensitivity plus specificity Cloglog threshold in Maxent. Colors correspond to the suitability score with dark blue (0) representing the most unsuitable places and red (1) representing the most suitable places. The figures were generated using Maxent ver. 3.4.1. (released under MIT license) and processed in QGis ver. 3.32 (licensed under Creative Commons Attribution-ShareAlike 3.0 license (CC BY-SA)).

*Lygocoris pabulinus*. The preferred conditions for this species during the LIG period covered large regions in the Palearctic and large areas above the polar circle in Europe and Asia (∼12 mln km^2^). During the LGM, this species was in at least two isolated refugia; one covered Southern Europe and the Caucasus, and the second was in East Asia (∼11–12 mln km^2^). During the MH, *Lygocoris pabulinus* most probably became trans-Palearctic again (11–14 mln km^2^), and its preferred conditions extended to the polar areas in Europe and Asia. The total area with suitable conditions now is larger than during the LIG (∼15 mln km^2^). It is expected that in the future, the distribution of this species will shift toward Asian regions, including its polar areas, and it will be less widespread in Europe. The projections predict that the area of preferred climatic conditions will not expand or will only slightly expand (MIROC-ESM for rcp26) for this species in the future (rcp26: 14–16 mln km^2^, rcp85: 11–13 mln km^2^); however, it will shift toward Asian regions (current: 8 mln km^2^, rcp26: 9–12 mln km^2^, rcp85: 9–10 mln km^2^) (Figs. 1 and 4, Figs. SI1, SI2, SI5, SI6).

**Figure 5:**
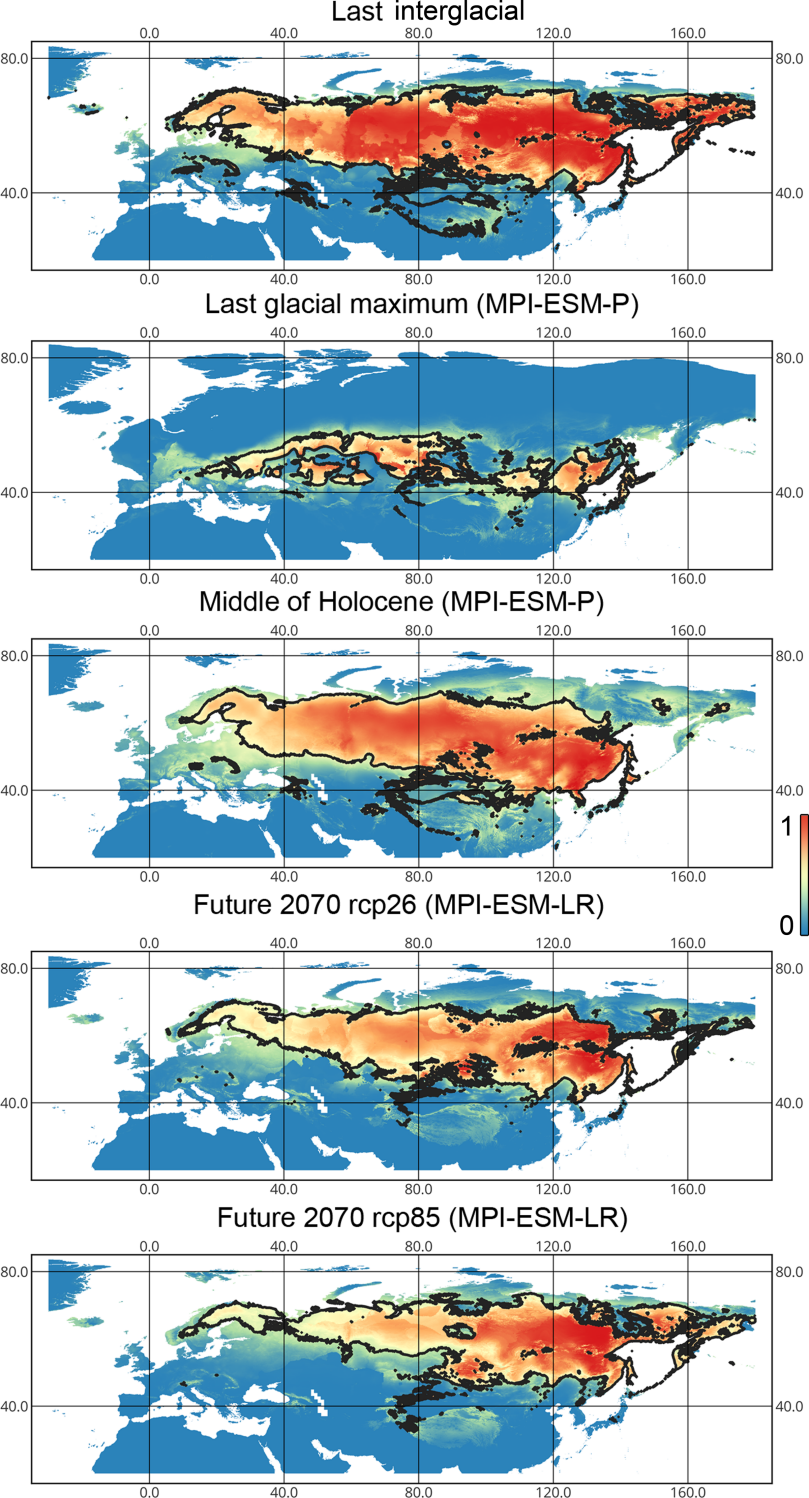
Geographic projections of the Maxent models to the past and future conditions using the MPI-ESM climate model for *Lygus punctatus*. The solid line corresponds to the maximum training sensitivity plus specificity Cloglog threshold in Maxent. Colors correspond to the suitability score with dark blue (0) represening the most unsuitable places and red (1) representing the most suitable places. The figures were generated using Maxent ver. 3.4.1. (released under MIT license) and processed in QGis ver. 3.32 (licensed under Creative Commons Attribution-ShareAlike 3.0 license (CC BY-SA)).

*Lygus punctatus*. During the LIG, the preferred climatic conditions for this species covered large areas in the Palearctic, including polar regions in Europe and Asia (∼20 mln km^2^). However, Central and Southern Europe were not suitable for this species. During the LGM, the refugia could be in Eastern Europe, Southern Siberia, Central Asia, and East Asia (∼2–7 mln km^2^). The climate model MIROC-ESM shows a narrower and more disrupted area with preferred conditions than the other two models. By the MH, this species most probably became widespread again, occupying a smaller area than during the LIG (∼12–18 mln km^2^), and today the area of preferred climatic conditions can be the same or narrower than during the MH (∼14 mln km^2^). The conditions in the polar regions in Asia in the Holocene until the 21st century were unsuitable for this species. It is expected that in the future, *Lygus punctatus* will expand its distribution to the polar regions in Asia (current: 0 mln km^2^, rcp26: 0.27–1.6 mln km^2^, rcp85: 1–1.9 mln km^2^) and narrow its distribution in Europe (current: 3.8 mln km^2^, rcp26: 1.6–2.6 mln km^2^, rcp85: 0.38–1.5 mln km^2^). The projections based on the CCSM4 and MPI-ESM climate models show that the total distribution area will not considerably expand for this species in the future and might even shrink with the high CO_2_ emission. The projection based on MIROC-ESM predicted area of preferred conditions growth for the rcp26 scenario and area decrease for the rcp85 scenario (rcp26: 13–16 mln km^2^, rcp85: 11–13 mln km^2^) (Figs. 1 and 5, Figs. SI1, SI2, SI7, SI8).

**Variables contributing to the climatic niches.** The sets of climatic variables strongly contributing to the models are different among the three species (Table SI2).

For *Lygus punctatus*, only temperature-related variables are important. Annual temperature (bio 1) and temperature seasonality (bio 4) has high PC and PI. Additionally, mean temperature of warmest quarter (bio 10) has high PI. The distribution of this species is shifted toward places with warm summers (bio 5, bio 10), warm wettest periods (bio 8), and colder winters (bio 6, bio 11). It also prefers places with low isothermality (bio 3), low annual precipitation (bio 12), and precipitation over the warmest, wettest, and driest periods (bio 13, 14, 16–18) (Fig. SI9).

In *Liocoris tripustulatus*, annual mean temperature (bio 1), precipitation of driest quarter (bio 17) and precipitation of coldest quarter (bio 19) have high PC and PI. Minimum temperature of coldest month (bio 6) also has high PI. Suitable conditions for this species are shifted to places with warmer summers and winters (bio 1, 5–11) and less pronounced seasonality (bio 4 and bio 7). It also lives in places with lower annual precipitation (bio 12) as well as precipitation over the wettest month, the wettest quarter, and the warmest month (bio 13, 16, 18) (Fig. SI9).

In *Lygocoris pabulinus*, annual precipitation (bio 12), precipitation of driest month (bio 14) and precipitation of the warmest quarter (bio 18) have high PC and PI. Mean temperature of driest quarter has a high PI. The climatic variables for this species generally have wider or similar ranges compared to those for *Lygus punctatus* and *Liocoris tripustulatus* (Fig. SI9).

**Species differentiation with PCA.** The PCA applied for the dataset with three species revealed four principal components (PCs) with nonzero eigenvalues. According to the broken-stick model, three of these components are deemed statistically significant. Despite this, ANOVA identified no significant differences between species in the analyses conducted on PC3. Therefore, we omit the statistical test results for this component in the discussion. The combined influence of the first two PCs accounts for 70.3% of the overall variation for the full dataset. The results for the complete and reduced datasets from ANOVA and Tukey’s test on the first two PCs show significant species differences across all analyses based on the climatic data, except for the results for Tukey’s test (full dataset) and the ANOVA (reduced dataset) on PC2 for the *Lygocoris pabulinus*–*Lygus punctatus* pair (Fig. SI10, Table SI3).

In all the analyses, PC1 has its highest loadings on the following climatic variables: temperature annual range (bio 7), temperature seasonality (bio 4), and minimum temperature of coldest month (bio 6). Similarly, PC2 displays its highest loadings on one common variable throughout all the analyses—namely, precipitation of warmest quarter (bio 18). However, the two other top variables vary. For the *Liocoris tripustulatus*–*Lygus punctatus* pair, they are the mean temperature of warmest quarter (bio 10) and maximum temperature of warmest month (bio 5), whereas in all other analyses (the full dataset, *Liocoris tripustulatus*–*Lygocoris pabulinus*, and *Lygocoris pabulinus*–*Lygus punctatus*), these variables are precipitation of wettest quarter (bio 16) and precipitation of wettest month (bio 13) (Fig. SI9, Table SI4). This distinction suggests that these variables are specific to species pairs. Given the highest input in the PC, these high-loading variables have a significant contribution to species differentiation.

**Figure 6:**
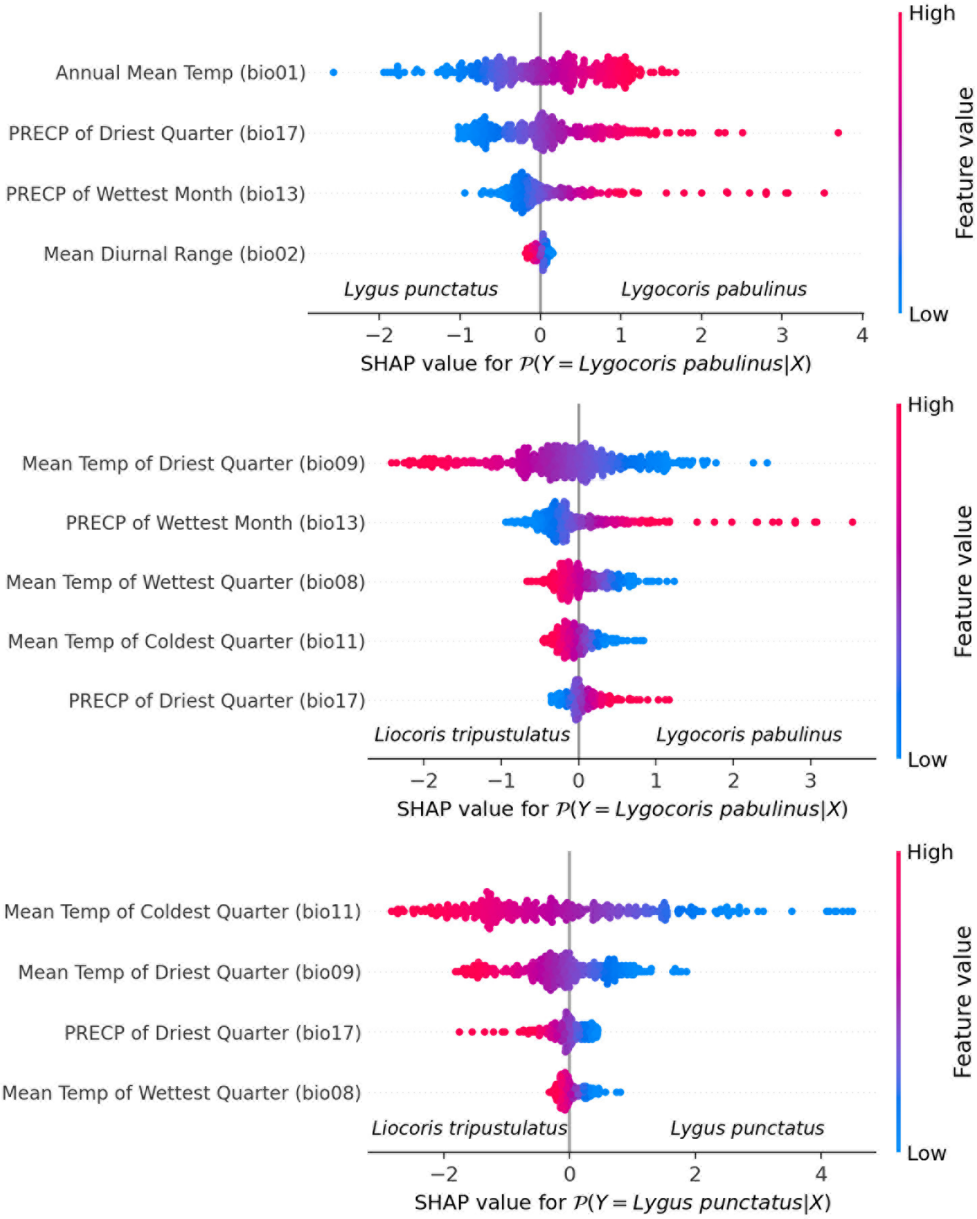
Beeswarm plot obtained with the logistic regression for the species pairs. Plots shows the variables highly contributing to the differences within the species pairs. Blue color means the lower variable values; red color means the higher variable values.

**Species differentiation with linear models.** We fit three separate logistic regression models for each species pair. The feature selection resulted in the following final lists of variables: for *Lygus punctatus*–*Lygocoris pabulinus*: annual mean temperature (bio 1), mean diurnal range (bio 2), precipitation of wettest month (bio 13), and precipitation of driest quarter (bio 17); for *Liocoris tripustulatus*–*Lygocoris pabulinus*: mean temperature of wettest quarter (bio 8), mean temperature of driest quarter (bio 9), mean temperature of coldest quarter (bio 11), precipitation of the wettest month (bio 13), and precipitation of the driest quarter (bio 17); and for *Liocoris tripustulatus*–*Lygus punctatus*: mean temperature of wettest quarter (bio 8), mean temperature of driest quarter (bio 9), mean temperature of coldest quarter (bio 11), and precipitation of driest quarter (bio 17) (Fig. 6, Table SI5).

The following coefficients were found as optimal for L2 penalty: *Lygus punctatus*–*Lygocoris pabulinus*: 0.293, *Liocoris tripustulatus*–*Lygocoris pabulinus*: 0.132, and *Liocoris tripustulatus*–*Lygus punctatus*: 0.212. The final ROC–AUC metrics on the test subset were as follows: *Lygus punctatus*–*Lygocoris pabulinus*: 0.80, *Liocoris tripustulatus*–*Lygocoris pabulinus:* 0.70, and *Liocoris tripustulatus*–*Lygus punctatus*: 0.96 (Fig. SI11, Table SI5).

For the *Lygus punctatus*–*Lygocoris pabulinus* pair, annual mean temperature (bio 1), precipitation of the wettest month (bio 13), and precipitation of the driest quarter (bio 17) are responsible for the direct dependency and mean diurnal range (bio 2) is responsible for the inverse dependency of the confidence that an observation belongs to *Lygocoris pabulinus*. For the *Liocoris tripustulatus*–*Lygocoris pabulinus* pair, precipitation of the wettest month (bio 13) and precipitation of the driest quarter (bio 17) are responsible for the direct dependency and mean temperature of wettest quarter (bio 8), mean temperature of driest quarter (bio 9), and mean temperature of coldest quarter (bio 11) are responsible for inverse dependency of the confidence that the observation belongs to *Lygocoris pabulinus*. For *Liocoris tripustulatus*–*Lygus punctatus*, all the variables are responsible for inverse dependency of the confidence that the observation belongs to *Lygus punctatus* (Fig. SI11, Table SI5).

## Discussion

**Niche dynamics** **with** **respect to the temperature change.** Even if global warming has a positive influence on widespread species, including pests, their distribution ranges might also be affected by non-climatic variables. However, areas of preferred climatic condition should be directly connected with temperature changes. This study showed that the area of preferred climatic conditions dynamics are different in all three examined species and do not correspond to the global temperature change in the Pleistocene. Our results demonstrate that the area of preferred conditions during the LIG is larger than during the MH and at the present time in *Lygus punctatus* only. In most cases, except for the MPI-ESM climate model for *Lygus punctatus*, the distribution area at the present time is larger than during the MH. Our study shows that the increase of the total distribution area in the future with respect to the current conditions can be predicted only for the Western Palearctic *Liocoris tripustualtus*, whereas in the two other species, the total distribution area can decrease at least in the scenario with the highest CO_2_ emission. Among the three studied species, only in *Liocoris tripustulatus*, the area of preferred climatic conditions in the future is much larger than during the LIG. Another finding is that the species with the boreal distribution (*Lygus punctatus*) and the trans-Palearctic species (*Lygocoris pabulinus*) will reduce their distribution in Europe in both scenarios for 2070. The decrease will be more pronounced in the case of higher CO_2_ emissions. All three species will be more widely distributed in Asia; however, the difference between the current state and the 2070 scenario is more pronounced for *Liocoris tripustulatus*. All three species will also expand their distribution to the polar regions, supporting the results of previous studies (*e.g.*, Parmesan et al., 1999; Gutierrez, Ponti & Cossu, 2009; Bebber, Ramotowski & Gurr, 2013).

Our findings have two consequences. First, global temperature fluctuations alone cannot be used to predict area changes of preferred climatic conditions for species that are adapted for variable climatic zones now (*e.g.*, Shrestha, 2019; Lehmann et al., 2020). The distribution dynamics differ in species with different distributions, meaning that some currently successful species will benefit from climate warming, while for others, climate with cooler temperatures might be more preferable. Our study shows that the species widely distributed in the Western Palearctic but not in polar areas might expand their distribution area soon, which was also shown for the pests *Diuraphis noxia* (Kurdjumov, 1913) and *Bemisia tabaci* (Gennadius, 1889) (Gilioli et al., 2014; Jing et al., 2023). Second, species distribution dynamics will differ depending on the region, which was also found in previous studies (Taylor & Hastings, 2005; Thackeray et al., 2016). *Lygus punctatus* and *Lygocoris pabulinus* will be more widespread and may become more serious pests in Asia. However, the climate will not be so suitable for them in Europe, and they might leave its southern and central areas in a few decades.

**Climatic variables limiting widespread species distribution.** Our study shows that the precipitation variables can be as important as the temperature variables, at least for *Liocoris tripustulatus* and *Lygocoris pabulinus*. This confirms previous suggestions that global warming alone cannot predict widespread species and pest distribution range changes.

Annual mean temperature (bio 1) highly contributes to the models of *Liocoris tripustulatus* and *Lygus punctatus*, and this was also found for other widespread plant bug species (Namyatova, 2020), meaning that the temperature can be important for distribution changes, at least in some widespread species. This variable highly contributes to the differences between all species pairs in the PCA and is highly correlated with the winter temperatures (bio 6, bio 11) in all the studied species. Therefore, the annual temperature in this case is mostly defined by the winter temperatures (Data SI2, Table SI4), and these variables also highly contribute to the differences between the species pairs in the PCA and logistic regression. Although it was shown that many insect species would benefit from warmer summers (Bale et al., 2002; Skendžić et al., 2021), in the studied species, the winter temperature might play a more important role.

Logistic regression shows that *Lygus punctatus* lives in places with colder winters than the other two species, and its survival rates can decrease because of the warmer temperature. It is usually stated that pests will benefit from temperature growth. However, for diapausing insects, climate change might affect their synchronization with the environment and host plants (Skendžić et al., 2021). In freeze-avoidant insects adapted to temperatures less than −30 °C, thaws in winter with the subsequent sudden temperature drop can reduce their survival rate (Delisle, Bernier-Cardou & Labrecque, 2022). It was demonstrated that a higher pre-winter temperature might also decrease the survival rate in diapausing insects (Toxopeus et al., 2021). The overwintering strategies and life cycles in *Lygocoris pabulinus* and *Lygus punctatus* may differ, and *Lygocoris pabulinus* evolved more flexible mechanisms than *Lygus punctatus*. Another hypothesis is that *Lygus punctatus* does not require colder winters but might be univoltine and has obligate diapause, which might increase its survival in winter. The trade-off of this strategy is that such species have slower population growth (Skendžić et al., 2021). At least two other Palearctic *Lygus* species that are more abundant in the central and southern parts of the Western Palearctic can have multivoltine cycles (Saulich & Musolin, 2020), and *Lygus punctatus* may be unable to compete with them in milder temperatures. *Lygocoris pabulinus* can also have more than one population per year, which might contribute to its wider distribution (Saulich & Musolin, 2020).

*Liocoris tripustulatus* prefers warmer winters than *Lygocoris pabulinus* and *Lygus punctatus*. Additionally, in both pairs with *Liocoris tripustulatus*, mean temperature of wettest quarter (bio 8) and mean temperature of driest quarter (bio 9) highly contribute to the differences among the species, and *Liocoris tripustualtus* prefers the higher temperatures. This suggests that in the case of *Liocoris tripustulatus*, lower temperatures, especially in winter, can prevent this species from spreading eastward and poleward. It was shown that in insects, survival significantly drops at temperatures below −25 °C (Dukes et al., 2009). However, among the three studied species, only *Liocoris tripustulatus* might have serious problems with survival rates at these temperatures. The specimens of this species were not found in places where minimum temperature of coldest monts (bio 6) was below −25.1 °C, whereas the two other species can survive temperatures well below −40 °C (Fig. SI9).

The logistic regression results show that *Lygocoris pabulinus* prefers places with higher precipitations than the other two species. This might be at least partly connected with the fact that the nymphs of this species develop on trees (Saulich & Musolin, 2020), which are usually less represented in areas with dry climates.

Precipitation of wettest month (bio 13) might also play an important role in the distribution of the studied species because it contributes to the differences in PCA and logistic regression in the species pairs comprising *Lygocoris pabulinus*. This variable highly correlates with precipitation of warmest quarter (bio 18) in *Lygocoris pabulinus* and *Lygus punctatus*. Logistic regression shows that *Lygus punctatus* lives in drier places in summer compared to *Lygocoris pabulinus*, and precipitation over this period might be a limiting factor for one or both species.

Precipitation of the driest quarter (bio 17) is another important variable because it can show the susceptibility of the species to drought. In the case of *Lygocoris pabulinus* and *Lygus punctatus*, it highly correlates with precipitation of the coldest quarter (bio 19), meaning that in most territories inhabited by these two species, the driest season is winter. In the case of *Liocoris tripustulatus*, it has a significant correlation with annual precipitation (bio 12) (PCor > 80%) and with precipitation of the warmest quarter (bio 18) (PCor > 70%). The logistic regression shows that bio17 has a high contribution to the differences within all species pairs. In the case of the *Lygocoris pabulinus*–*Lygus punctatus* pair, the latter can live in places with lower precipitation over the driest (and coldest) periods, which might mean that *Lygocoris pabulinus* needs higher snow cover for survival. This supports the importance of higher temperatures for the survival of *Lygocoris pabulinus* during the winter as snow cover can protect these insects from the cold. *Liocoris tripustulatus* needs less precipitation over the driest quarter than *Lygocoris pabulinus* and more precipitation than *Lygus punctatus.* Given that *Lygocoris pabulinus* does not inhabit Mediterranean regions and steppes, the area increase of the corresponding biomes might negatively affect the distribution of this species. A high level of precipitation in summer might not be suitable for *Liocoris tripustulatus*, and this might also explain why it is more widespread in temperate and Southern European areas now rather than in Northern European areas and Siberia.

**Study limitations and future directions.** One of the main tasks for ENM is to create a valid dataset, and the localities used for the analyses should cover the largest part of the species’ distribution area. Although we obtained the localities from different sources, there could still be areas in the Palearctic that were not covered for some or all of the studied species. Our study was based on the ZISP collection, which hosts numerous specimens from different Russian regions as well as other countries. However, some large Siberian regions remain hard to reach, and plant bugs were not collected from there. Additionally, *Lygus punctatus* was recorded from Central and Western Europe, and this was not reflected in the present dataset. For this work and the previous study (Namyatova, Tyts & Bolshakova, 2023), we examined numerous specimens of this genus from Europe preserved at ZISP and collected by us and our colleagues and did not find any specimens of *Lygus punctatus* from Central and Southern Europe. So far, the data indicates that this species is mostly boreal. It might still inhabit Central and Eastern Europe, but most probably, it is very rare there.

Our study shows that climate change affects widely distributed Palearctic species in different ways. However, there might be patterns depending on the species’ other environmental preferences, sizes, and biologies, which are yet unknown. There are many more widely distributed species in Miridae and other insect groups, and they should also be studied to further investigate how climate change affects the relationships among the species’ distribution and climate change.

## Conclusions

In this study, the area of preferred climatic conditions dynamics of three plant bug species distributed in Europe and Asia were compared. We investigated how temperature rise might influence the distribution of these taxa and whether it is beneficial for the widely distributed pest species. The variables contributing to the climatic models and the distribution differences within the species pairs were revealed. The results show that all three species have different areas of preferred climatic condition dynamics and that temperature rise is not necessarily beneficial for the widely distributed species in the Palearctic. Geographic projections of the climatic models for different periods of all the studied taxa did not show a direct dependency on temperature changes. Our results show that the distributions of *Lygocoris pabulinus* and *Lygus punctatus* can even shrink soon, especially in Europe. In the future, the distribution areas of all the three species can increase in Asia and the polar regions. Annual mean temperature and temperatures in winter most probably shape the distribution of *Liocoris tripustulatus* and *Lygus punctatus* but in different ways. *Liocoris tripustulatus* prefers warmer areas, whereas *Lygus punctatus* can be found more often in colder places than the other two species. Different precipitation variables also contribute to the models of *Liocoris tripustulatus* and *Lygocoris pabulinus* as well as the differences between the species pairs. Therefore, they might also be important factors limiting the distribution of the widely distributed species. In the future, studies of more widespread Palearctic species from different groups are needed to better understand the influence of climate on their distribution.

## Supplemental Information

10.7717/peerj.18377/supp-1 Supplemental Information 1 Supplementary data Data SI1: Locality and collection event data for the *Liocoris tripistulatus*, *Lygocoris pabulinus* and *Lygus punctatus* used for the ecological niche modelling, PCA, and logistic regression. Data SI2. Pearson’s correlations of the variables. The high correlations (<90%) are highlighted in red. Data SI3. Areas calculated from the geographic projections of the ecological niche models for each species, dataset, and climate model (km2).

10.7717/peerj.18377/supp-2 Supplemental Information 2 Graphs showing the areas of preferred climatic conditions dynamics in time X-axis correspond to the different time periods. Y-axis correspond to the area of preferred conditions. Based on the Maxent models, calculated with CR dataset and CCSM4 climate model.For the future, green line corresponds to the rcp85 scenario (the highest CO_2_ emission), and dark blue line corresponds to the rcp26 scenario (the lowest CO_2_ emission).

10.7717/peerj.18377/supp-3 Supplemental Information 3 Graphs showing the areas of preferred climatic conditions dynamics in time X-axis correspond to the different time periods. Y-axis correspond to the area of preferred conditions. Based on the Maxent models, calculated with CR dataset and MIROC-ESM climate model. For the future, green line corresponds to the rcp85 scenario (the highest CO_2_ emission), and dark blue line corresponds to the rcp26 scenario (the lowest CO_2_ emission).

10.7717/peerj.18377/supp-4 Supplemental Information 4 Geographical projections of the Maxent models for the past and future conditions obtained using the CCSM4 climate model for *Liocoris tripustulatus* The solid line corresponds to the maximum training sensitivity plus specificity Cloglog threshold in Maxent. Colors correspond to the suitability score with dark blue (0) representing the most unsuitable places and red (1) representing the most suitable places. The figures were generated using Maxent ver. 3.4.1. (released under MIT license) and processed in QGis ver. 3.32 (licensed under Creative Commons Attribution-ShareAlike 3.0 license (CC BY-SA).

10.7717/peerj.18377/supp-5 Supplemental Information 5 Geographical projections of the Maxent models for the past and future conditions obtained using the MIROC-ESM climate model for *Liocoris tripustulatus* The solid line corresponds to the maximum training sensitivity plus specificity Cloglog threshold in Maxent. Colors correspond to the suitability score with dark blue (0) representing the most unsuitable places and red (1) representing the most suitable places. The figures were generated using Maxent ver. 3.4.1. (released under MIT license) and processed in QGis ver. 3.32 (licensed under Creative Commons Attribution-ShareAlike 3.0 license (CC BY-SA).

10.7717/peerj.18377/supp-6 Supplemental Information 6 Geographical projections of the Maxent models for the past and future conditions obtained using the CCSM4 climate model for *Lygocoris pabulinus* The solid line corresponds to the maximum training sensitivity plus specificity Cloglog threshold in Maxent. Colors correspond to the suitability score with dark blue (0) representing the most unsuitable places and red (1) representing the most suitable places. The figures were generated using Maxent ver. 3.4.1. (released under MIT license) and processed in QGis ver. 3.32 (licensed under Creative Commons Attribution-ShareAlike 3.0 license (CC BY-SA).

10.7717/peerj.18377/supp-7 Supplemental Information 7 Geographical projections of the Maxent models for the past and future conditions obtained using the MIROC-ESM climate model for *Lygocoris pabulinus* The solid line corresponds to the maximum training sensitivity plus specificity Cloglog threshold in Maxent. Colors correspond to the suitability score with dark blue (0) representing the most unsuitable places and red (1) representing the most suitable places. The figures were generated using Maxent ver. 3.4.1. (released under MIT license) and processed in QGis ver. 3.32 (licensed under Creative Commons Attribution-ShareAlike 3.0 license (CC BY-SA).

10.7717/peerj.18377/supp-8 Supplemental Information 8 Geographical projections of the Maxent models for the past and future conditions obtained using the CCSM4 climate model for *Lygus punctatus* The solid line corresponds to the maximum training sensitivity plus specificity Cloglog threshold in Maxent. Colors correspond to the suitability score with dark blue (0) representing the most unsuitable places and red (1) representing the most suitable places. The figures were generated using Maxent ver. 3.4.1. (released under MIT license) and processed in QGis ver. 3.32 (licensed under Creative Commons Attribution-ShareAlike 3.0 license (CC BY-SA).

10.7717/peerj.18377/supp-9 Supplemental Information 9 Geographical projections of the Maxent models for the past and future conditions obtained using the MIROC-ESM climate model for *Lygus punctatus* The solid line corresponds to the maximum training sensitivity plus specificity Cloglog threshold in Maxent. Colors correspond to the suitability score with dark blue (0) representing the most unsuitable places and red (1) representing the most suitable places. The figures were generated using Maxent ver. 3.4.1. (released under MIT license) and processed in QGis ver. 3.32 (licensed under Creative Commons Attribution-ShareAlike 3.0 license (CC BY-SA).

10.7717/peerj.18377/supp-10 Supplemental Information 10 The graphs, showing the climate variable ranges for each species “Model” - ranges obtained from the Maxent models, “record” - ranges obtained from the species records used in this study.

10.7717/peerj.18377/supp-11 Supplemental Information 11 PCA results

10.7717/peerj.18377/supp-12 Supplemental Information 12 ROC curves and AUC values resulted from the logistic regression

10.7717/peerj.18377/supp-13 Supplemental Information 13 All sets of parameters and variables, used for the niche modeling, with corresponding AUC values, partial ROC values, and omission rates Omission rate corresponds to “10 percentile training presence test omission” in Maxent results. The sets of parameters and variables used for the visualization and niche comparison are in bold.

10.7717/peerj.18377/supp-14 Supplemental Information 14 Variables, contributing to the ENMs of three studied species The variables used for modeling are marked with “X”. PC and PI denote the variables having PC and PI higher than 10%.

10.7717/peerj.18377/supp-15 Supplemental Information 15 Results of ANOVA for all datasets and Tukey’s tests for the full dataset, p-values ¡0.001 are highlighted in gray

10.7717/peerj.18377/supp-16 Supplemental Information 16 Loadings from the PCA analysis The highest three loadings for each PC are marked in bold.

10.7717/peerj.18377/supp-17 Supplemental Information 17 Log regression coefficients for the logistic regression

## Additional Information and Declarations

### DNA Deposition

The following information was supplied regarding the deposition of DNA sequences: The cytochtome c oxydase subunite I sequences are available at Genbank: OR855716–OR855732, OR864695–OR864727, OR855736–OR855741.

### Data Availability

The following information was supplied regarding data availability: The scripts prepared for this study are available at Github and Zenodo: - https://github.com/vtyts/ThreeMirini
- Tyts, V., & Popkov, A. (2024). Climate change effect on the widely distributed Palearctic plant bug species (Insecta: Heteroptera: Miridae). Zenodo. https://doi.org/10.5281/zenodo.13348220
