# Supplementary figures and images for: Climate change effect on the widely distributed Palearctic plant bug species (Insecta: Heteroptera: Miridae)
Source: PeerJ. 2024 Nov 22;12:e18377. doi: 10.7717/peerj.18377 (PMC11587874; doi:10.7717/peerj.18377)

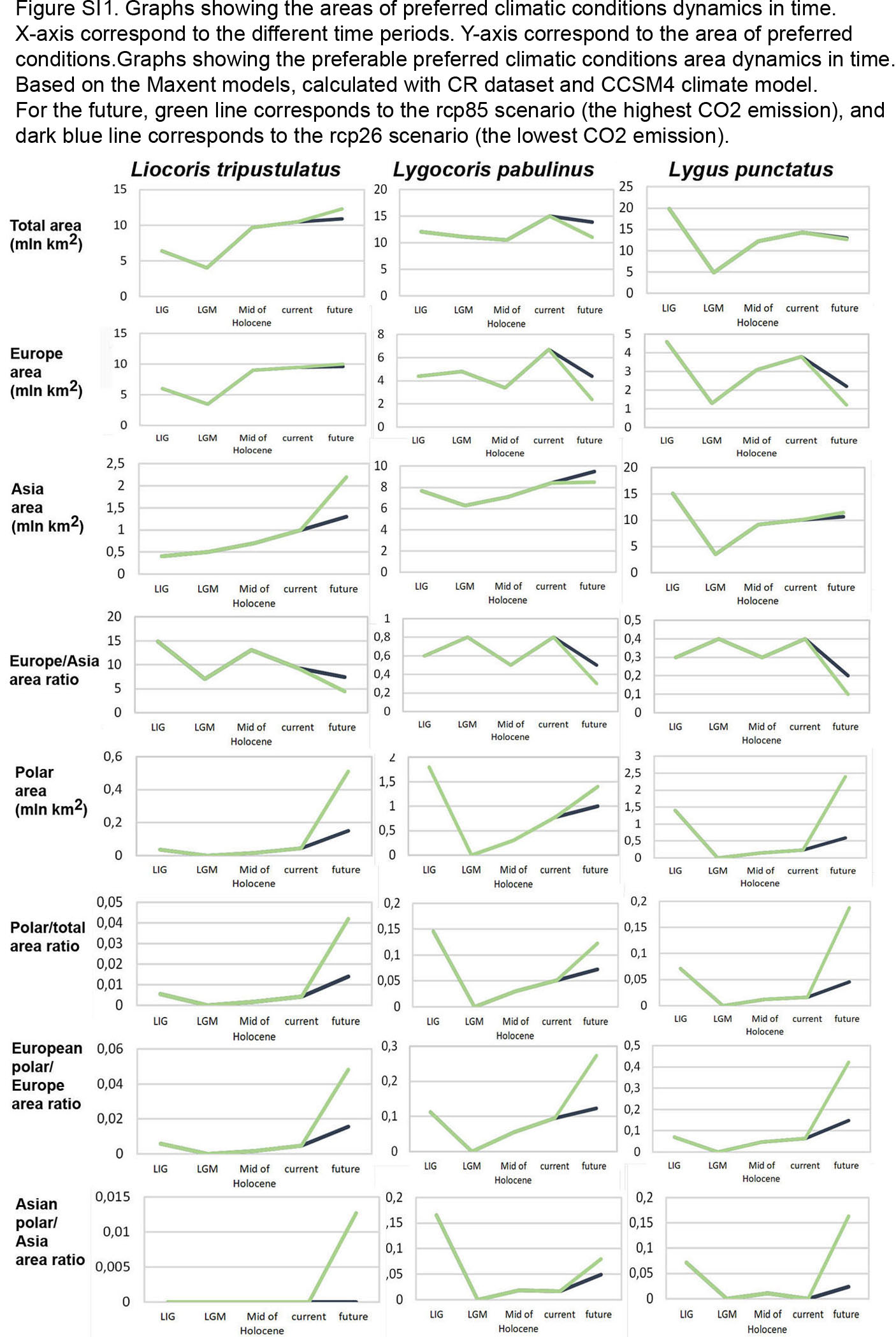

Supplement: Supplemental Information 2 — X-axis correspond to the different time periods. Y-axis correspond to the area of preferred conditions. Based on the Maxent models, calculated with CR dataset and CCSM4 climate model.For the future, green line corresponds to the rcp85 scenario (the highest CO2 emission), and dark blue line corresponds to the rcp26 scenario (the lowest CO2 emission). [file peerj-12-18377-s002.jpg]

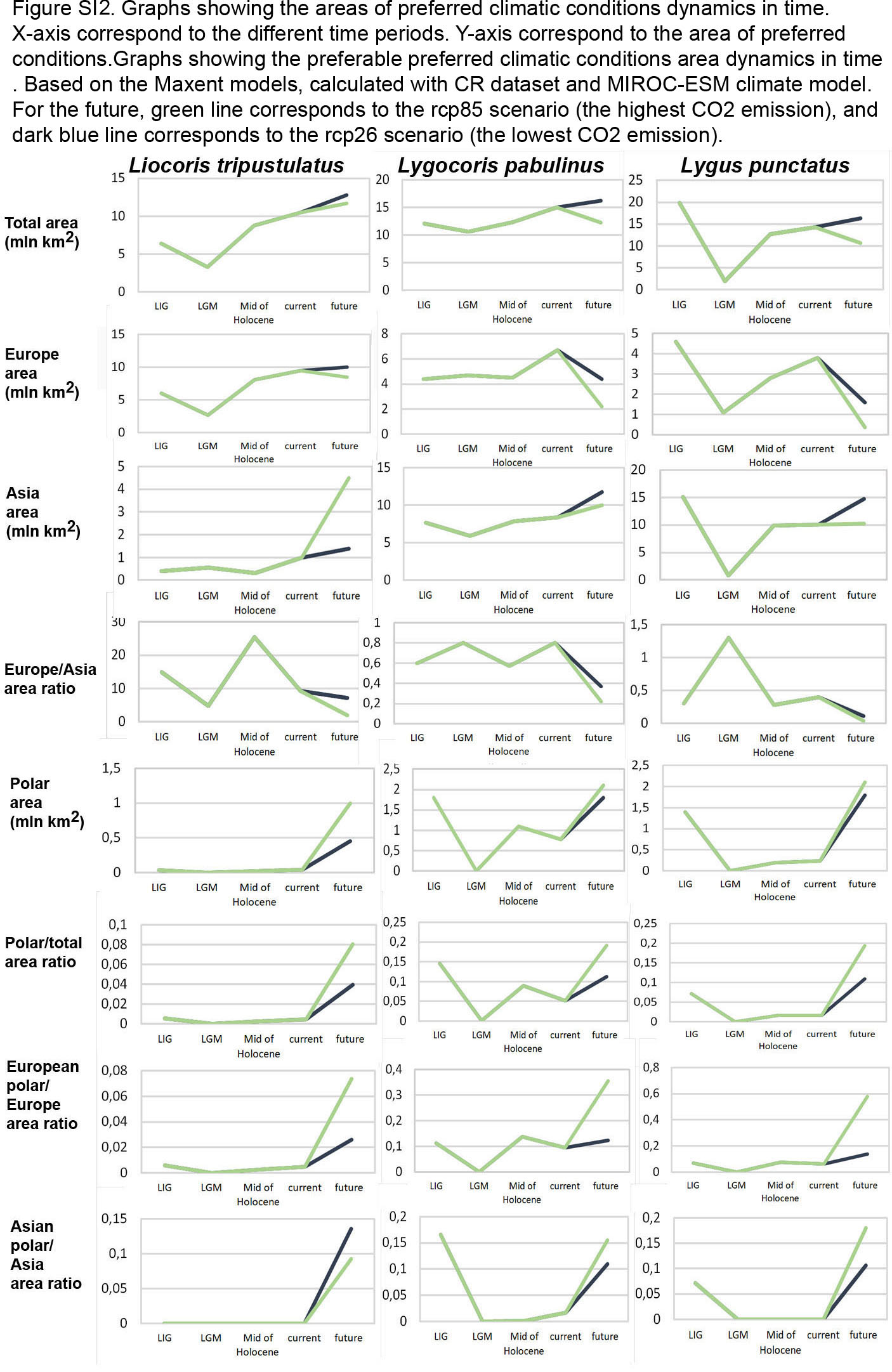

Supplement: Supplemental Information 3 — X-axis correspond to the different time periods. Y-axis correspond to the area of preferred conditions. Based on the Maxent models, calculated with CR dataset and MIROC-ESM climate model. For the future, green line corresponds to the rcp85 scenario (the highest CO2 emission), and dark blue line corresponds to the rcp26 scenario (the lowest CO2 emission). [file peerj-12-18377-s003.jpg]

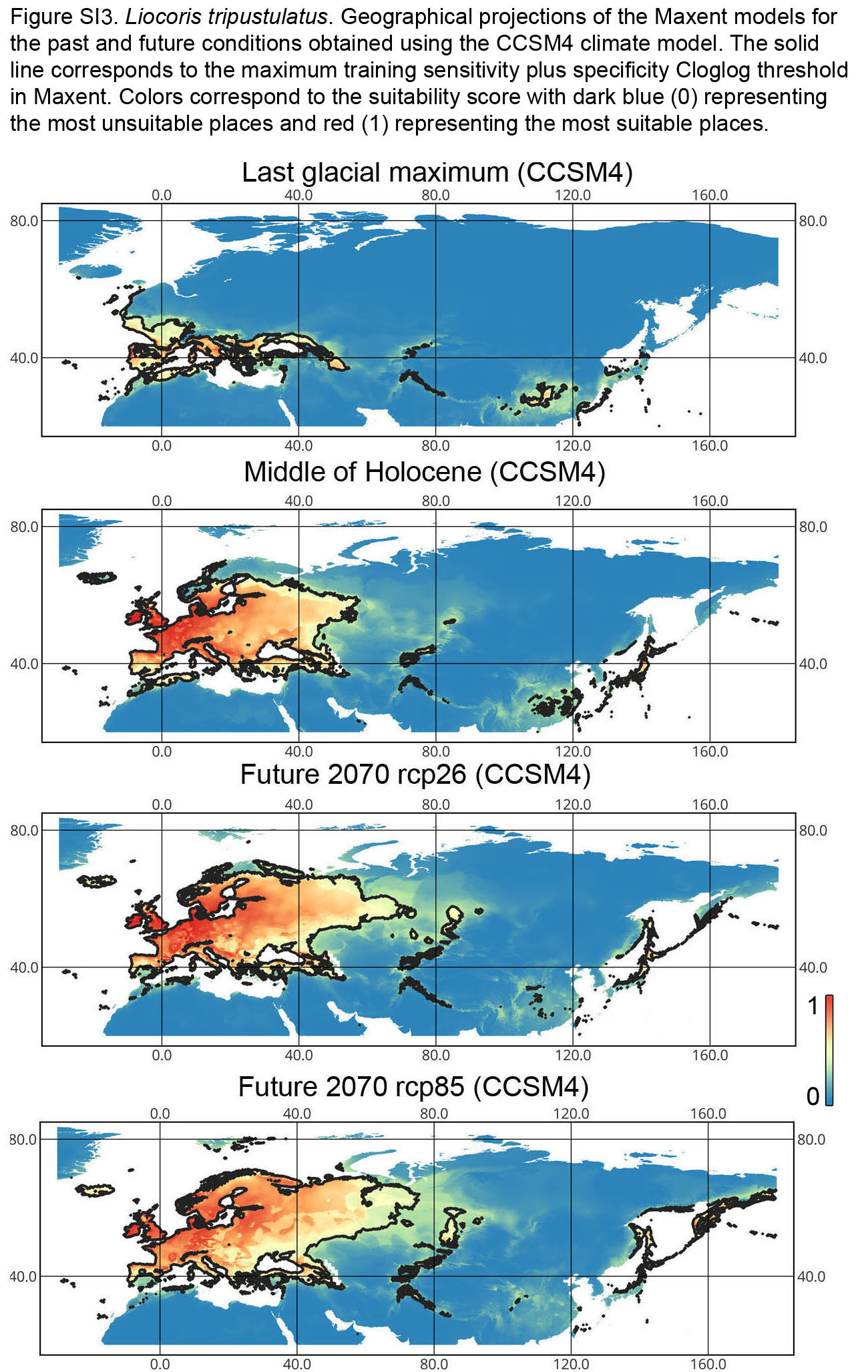

Supplement: Supplemental Information 4 — The solid line corresponds to the maximum training sensitivity plus specificity Cloglog threshold in Maxent. Colors correspond to the suitability score with dark blue (0) representing the most unsuitable places and red (1) representing the most suitable places. The figures were generated using Maxent ver. 3.4.1. (released under MIT license) and processed in QGis ver. 3.32 (licensed under Creative Commons Attribution-ShareAlike 3.0 license (CC BY-SA). [file peerj-12-18377-s004.jpg]

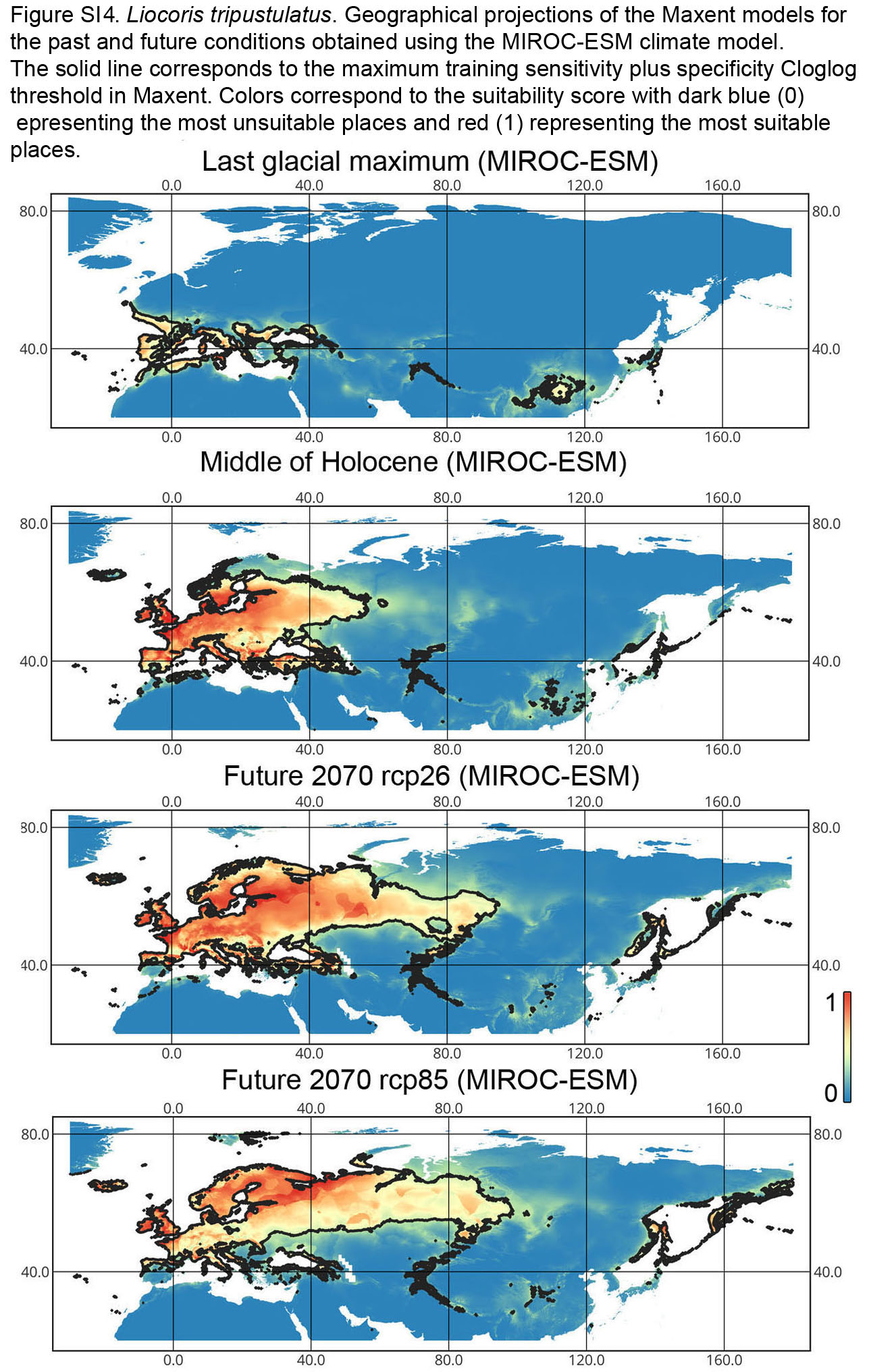

Supplement: Supplemental Information 5 — The solid line corresponds to the maximum training sensitivity plus specificity Cloglog threshold in Maxent. Colors correspond to the suitability score with dark blue (0) representing the most unsuitable places and red (1) representing the most suitable places. The figures were generated using Maxent ver. 3.4.1. (released under MIT license) and processed in QGis ver. 3.32 (licensed under Creative Commons Attribution-ShareAlike 3.0 license (CC BY-SA). [file peerj-12-18377-s005.jpg]

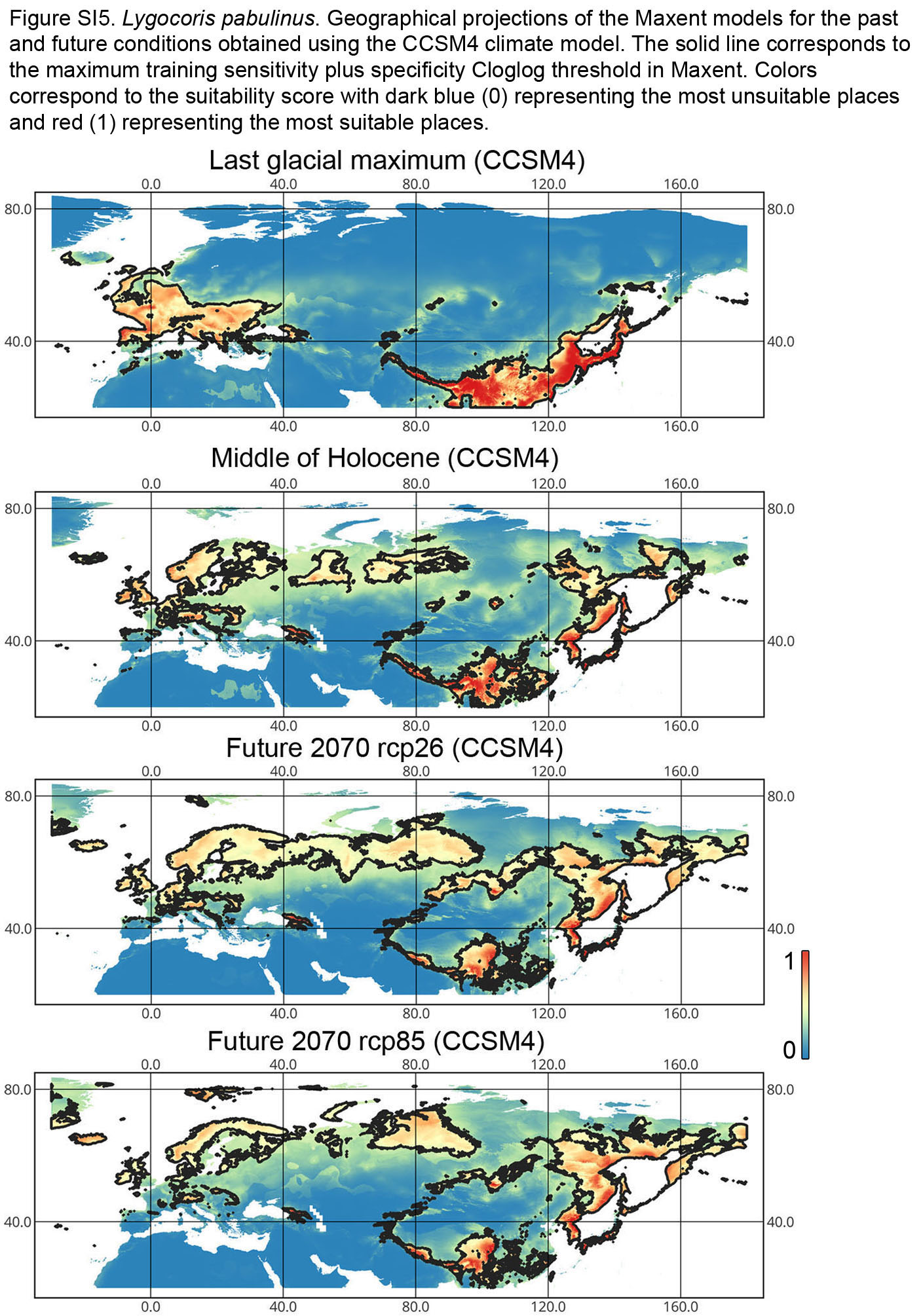

Supplement: Supplemental Information 6 — The solid line corresponds to the maximum training sensitivity plus specificity Cloglog threshold in Maxent. Colors correspond to the suitability score with dark blue (0) representing the most unsuitable places and red (1) representing the most suitable places. The figures were generated using Maxent ver. 3.4.1. (released under MIT license) and processed in QGis ver. 3.32 (licensed under Creative Commons Attribution-ShareAlike 3.0 license (CC BY-SA). [file peerj-12-18377-s006.jpg]

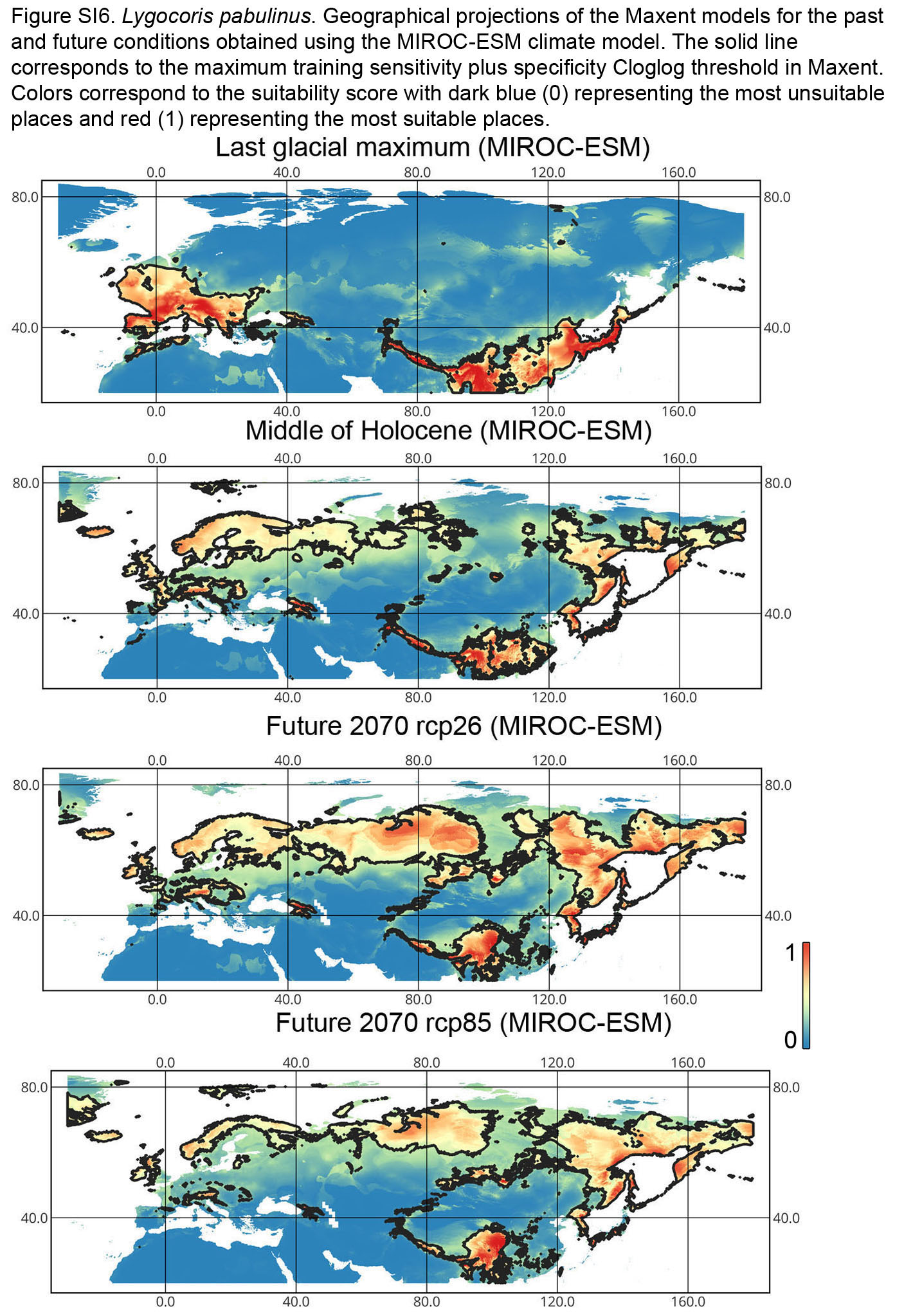

Supplement: Supplemental Information 7 — The solid line corresponds to the maximum training sensitivity plus specificity Cloglog threshold in Maxent. Colors correspond to the suitability score with dark blue (0) representing the most unsuitable places and red (1) representing the most suitable places. The figures were generated using Maxent ver. 3.4.1. (released under MIT license) and processed in QGis ver. 3.32 (licensed under Creative Commons Attribution-ShareAlike 3.0 license (CC BY-SA). [file peerj-12-18377-s007.jpg]

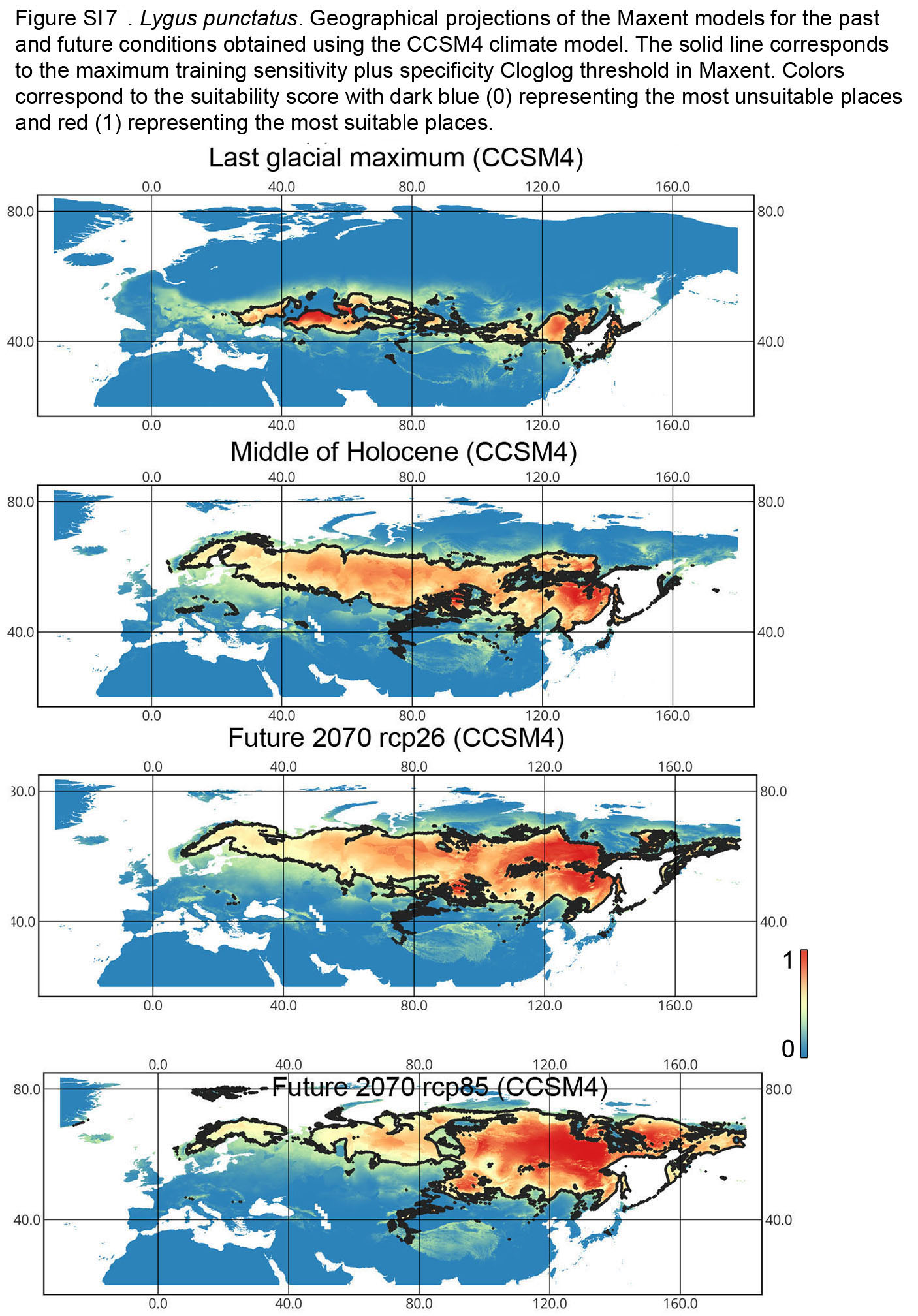

Supplement: Supplemental Information 8 — The solid line corresponds to the maximum training sensitivity plus specificity Cloglog threshold in Maxent. Colors correspond to the suitability score with dark blue (0) representing the most unsuitable places and red (1) representing the most suitable places. The figures were generated using Maxent ver. 3.4.1. (released under MIT license) and processed in QGis ver. 3.32 (licensed under Creative Commons Attribution-ShareAlike 3.0 license (CC BY-SA). [file peerj-12-18377-s008.jpg]

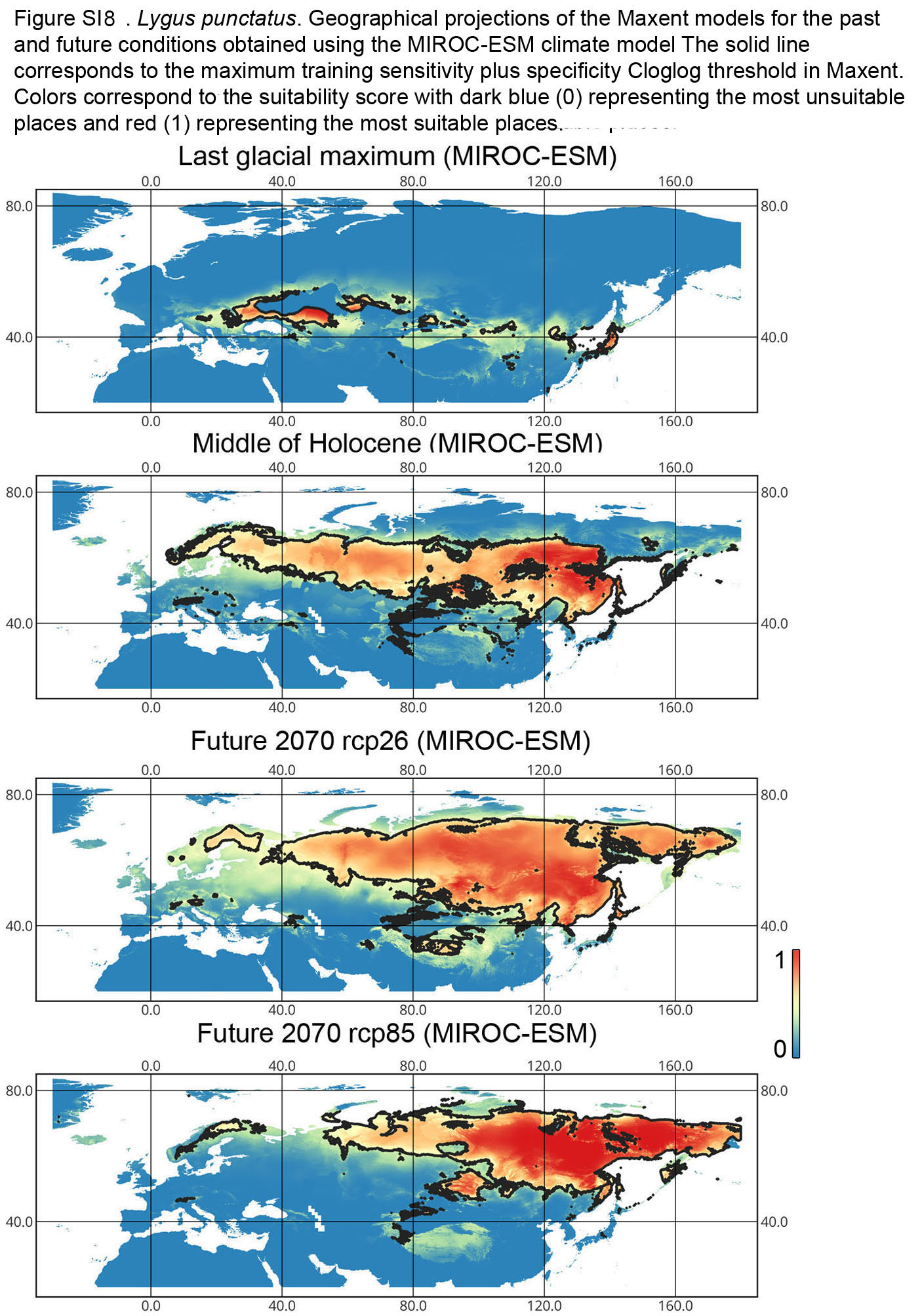

Supplement: Supplemental Information 9 — The solid line corresponds to the maximum training sensitivity plus specificity Cloglog threshold in Maxent. Colors correspond to the suitability score with dark blue (0) representing the most unsuitable places and red (1) representing the most suitable places. The figures were generated using Maxent ver. 3.4.1. (released under MIT license) and processed in QGis ver. 3.32 (licensed under Creative Commons Attribution-ShareAlike 3.0 license (CC BY-SA). [file peerj-12-18377-s009.jpg]

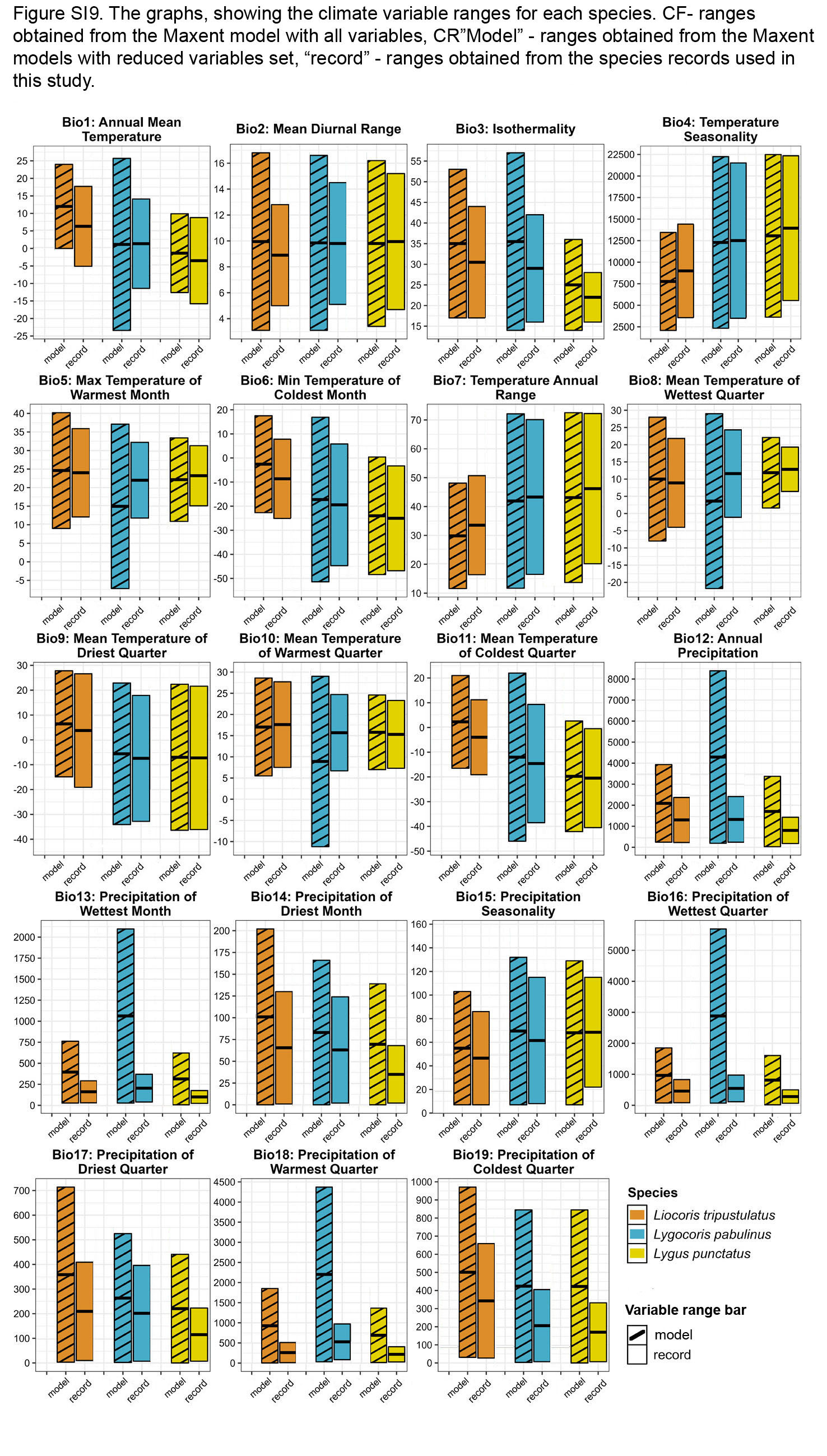

Supplement: Supplemental Information 10 — “Model” - ranges obtained from the Maxent models, “record” - ranges obtained from the species records used in this study. [file peerj-12-18377-s010.jpg]

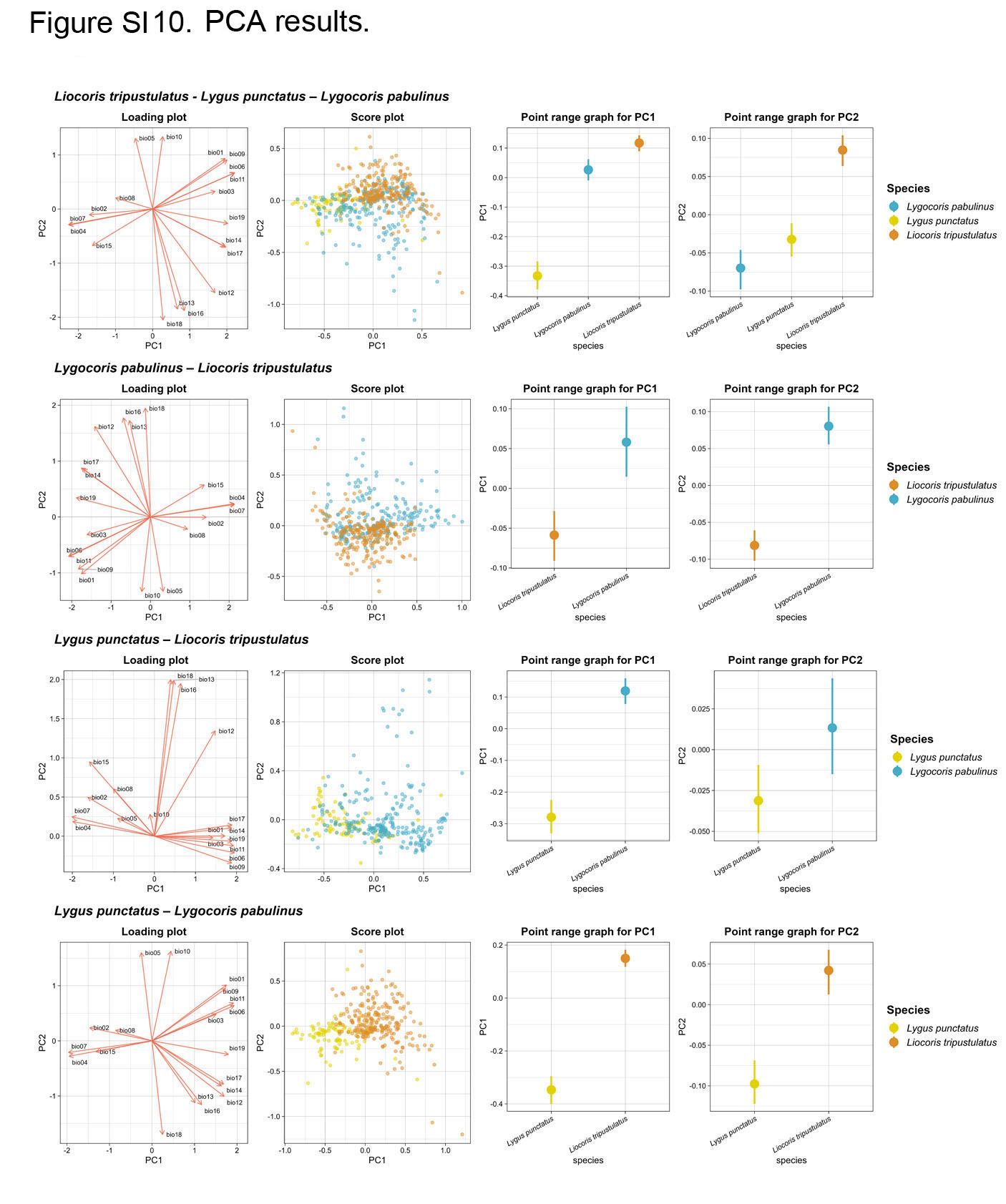

Supplement: Supplemental Information 11 [file peerj-12-18377-s011.jpg]

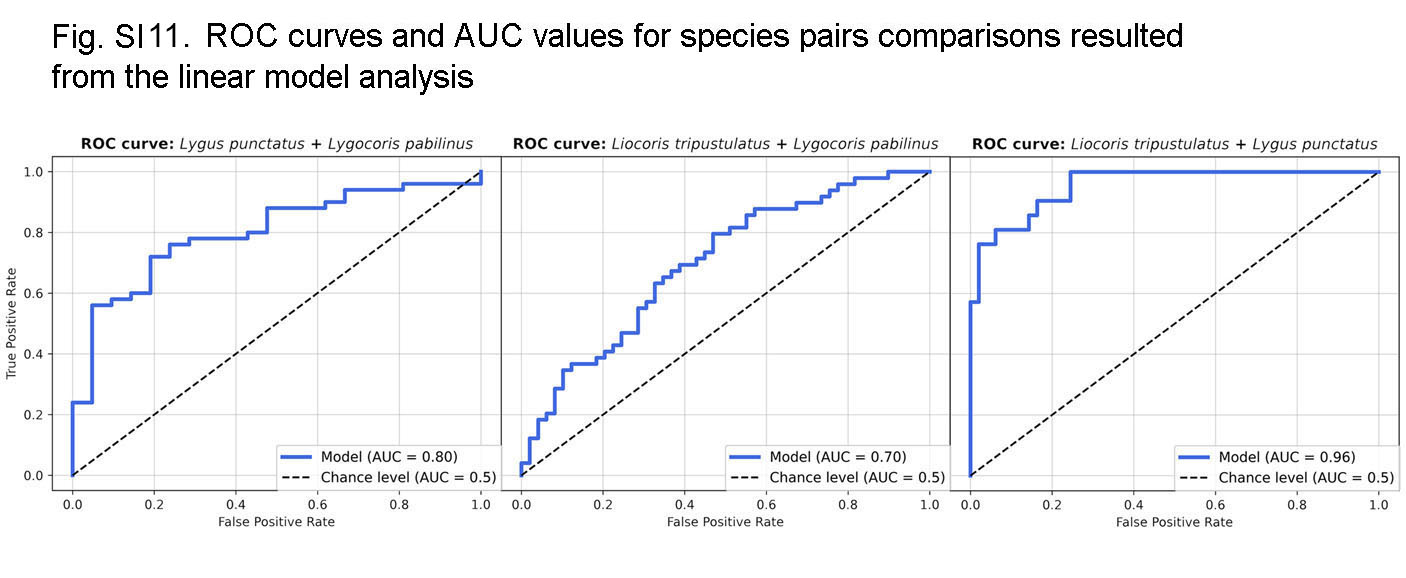

Supplement: Supplemental Information 12 [file peerj-12-18377-s012.jpg]
