# Supplementary material for: Climate change effect on the widely distributed Palearctic plant bug species (Insecta: Heteroptera: Miridae)
Source: PeerJ. 2024 Nov 22;12:e18377. doi: 10.7717/peerj.18377 (PMC11587874; doi:10.7717/peerj.18377)
Supplement: Supplemental Information 13 — Omission rate corresponds to “10 percentile training presence test omission” in Maxent results. The sets of parameters and variables used for the visualization and niche comparison are in bold. [file peerj-12-18377-s013.docx]

Table SI1. All sets of parameters and variables, used for the niche modelling, with corresponding AUC values, partial ROC values and omission rates. Omission rate corresponds to “10 percentile training presence test omission” in Maxent results. The sets of parameters and variables used for the visualization and niche comparison are in bold.

| species | parameters | variables | Partial ROC | AUC (Training) | AUC (Test) | AUC difference | Omission rate |
| --- | --- | --- | --- | --- | --- | --- | --- |
| *Lygocoris pabulinus* | L6 | 2–3, 5, 7–8, 12, 15, 18–19 | 0.743 | 0.7414 | 0.7389 | 0.0025 | 0.1803 |
| *Lygocoris pabulinus* | LQ1 | 1–3, 5, 7–9, 12, 15, 18–19 | 0.821 | 0.8133 | 0.7975 | 0.0158 | 0.1557 |
| *Lygocoris pabulinus* | LQH1 | 1–5, 8–9, 12, 14–15, 18 | 0.872 | 0.8776 | 0.8563 | 0.0213 | 0.1574 |
| *Lygocoris pabulinus* | H3 | 5, 9, 12, 14–15, 18 | 0.84 | 0.8370 | 0.8229 | 0.0141 | 0.1590 |
| ***Lygocoris pabulinus*** | **LQHP1.5** | **1–5, 8, 9, 12, 14**–**15, 18** | **0.855** | **0.8606** | **0.8339** | **0.0267** | **0.1541** |
| *Lygocoris pabulinus* | LQHPT1.5 | 1–5, 8–9, 12, 15–17 | 0.889 | 0.8885 | 0.8499 | 0.0386 | 0.1852 |
| *Liocoris tripustulatus* | H2.5 | 1–2, 4–6, 8, 12, 15, 17 | 0.922 | 0.9152 | 0.9106 | 0.0046 | 0.0934 |
| *Liocoris tripustulatus* | L6 | 2, 7, 8, 11, 13, 15, 19 | 0.865 | 0.8588 | 0.8496 | 0.0092 | 0.1933 |
| *Liocoris tripustulatus* | LQ6 | 1–3, 6–9, 13, 15, 19 | 0.902 | 0.8924 | 0.8858 | 0.0069 | 0.1117 |
| *Liocoris tripustulatus* | LQH2.5 | 1–3, 5–9, 12, 15, 17–19 | 0.923 | 0.9229 | 0.9071 | 0.0158 | 0.1000 |
| *Liocoris tripustulatus* | LQHP5.5 | 2–3, 7–11, 16–19 | 0.918 | 0.9167 | 0.9095 | 0.0072 | 0.0950 |
| ***Liocoris tripustulatus*** | **LQHPT5** | **1–3, 6–10, 15–19** | **0.924** | **0.9222** | **0.9140** | **0.0082** | **0.0767** |
| *Lygus punctatus* | L6 | 1–2, 8, 10, 12, 16 | 0.709 | 0.6998 | 0.7125 | 0.0127 | 0.1692 |
| ***Lygus punctatus*** | **LQ1.5** | **1–4, 8–10, 12, 15–16** | **0.883** | **0.8907** | **0.8756** | **0.0151** | **0.0769** |
| *Lygus punctatus* | LQH1 | 1–4, 8–10, 15, 18–19 | 0.923 | 0.9326 | 0.9132 | 0.0194 | 0.1077 |
| *Lygus punctatus* | H2.5 | 2, 7, 10, 13, 15, 19 | 0.893 | 0.9056 | 0.897 | 0.0086 | 0.1154 |
| *Lygus punctatus* | LQHP5 | – | - | N/A | N/A | N/A | N/A |
| *Lygus punctatus* | LQHPT5 | – | - | N/A | N/A | N/A | N/A |
