# Supplementary material for: Climate change effect on the widely distributed Palearctic plant bug species (Insecta: Heteroptera: Miridae)
Source: PeerJ. 2024 Nov 22;12:e18377. doi: 10.7717/peerj.18377 (PMC11587874; doi:10.7717/peerj.18377)
Supplement: Supplemental Information 14 — The variables used for modeling are marked with “X”. PC and PI denote the variables having PC and PI higher than 10%. [file peerj-12-18377-s014.docx]

Table SI2. The variables used for modelling are marked with ``X''. PC and PI denote the variables having PC and PI higher than 10%

|  | *Lygocoris pabulinus* | *Liocoris tripustulatus* | *Lygus punctatus* |
| --- | --- | --- | --- |
| Bio01 | X | PC PI | PC PI |
| Bio02 | X | X | X |
| Bio03 | X | X | X |
| Bio04 | Excluded | Excluded | PC PI |
| Bio05 | Excluded | Excluded | Excluded |
| Bio06 | Excluded | PI | Excluded |
| Bio07 | X | X | Excluded |
| Bio08 | X | X | X |
| Bio09 | PI | X | X |
| Bio10 | X | X | PI |
| Bio11 | Excluded | Excluded | Excluded |
| Bio12 | PC PI | Excluded | X |
| Bio13 | Excluded | Excluded | Excluded |
| Bio14 | PC PI | Excluded | Excluded |
| Bio15 | X | PI | X |
| Bio16 | Excluded | X | X |
| Bio17 | Excluded | PC | Excluded |
| Bio18 | PC PI | X | Excluded |
| Bio19 | Excluded | PC | Excluded |
