# Supplementary material for: Climate change effect on the widely distributed Palearctic plant bug species (Insecta: Heteroptera: Miridae)
Source: PeerJ. 2024 Nov 22;12:e18377. doi: 10.7717/peerj.18377 (PMC11587874; doi:10.7717/peerj.18377)
Supplement: Supplemental Information 15 [file peerj-12-18377-s015.docx]

Table SI3. Results of ANOVA for all datasets and Tukey’s tests for the full dataset, p-values <0.001 are highlighted in gray

| **Sp-sp** | **Tukey’s test (full dataset)** | | | **ANOVA (reduced datasets for pairs)** | | |
| --- | --- | --- | --- | --- | --- | --- |
|  | **PC1 padj** | **PC2 padj** | **PC3 padj** | **PC1 Pr(>F)** | **PC2 Pr(>F)** | **PC3 Pr(>F)** |
| ***Lygocoris pabulinus – Liocoris tripustulatus*** | 0.0002576 | 0.0000000 | 0.0116251 | 0.0000143 | 0.0000000 | 0.02132 |
| ***Lygus punctatus – Liocoris tripustulatus*** | 0.0000000 | 0.0000001 | 0.0322106 | 0.0000000 | 0.0000000 | 0.005992 |
| ***Lygus punctatus – Lygocoris pabulinus*** | 0.0000000 | 0.1610688 | 0.9539213 | 0.0000000 | 0.06888 | 0.4811 |
| **ANOVA Pr(>F)** | 0.0000000 | 0.0000000 | 0.005296 | – | – | – |
