# Supplementary material for: Climate change effect on the widely distributed Palearctic plant bug species (Insecta: Heteroptera: Miridae)
Source: PeerJ. 2024 Nov 22;12:e18377. doi: 10.7717/peerj.18377 (PMC11587874; doi:10.7717/peerj.18377)
Supplement: Supplemental Information 16 — The highest three loadings for each PC are marked in bold. [file peerj-12-18377-s016.docx]

Table SI4. Loadings from the PCA analysis. The highest three loadings for each PC are marked in bold

| **Var.** | **Full dataset** | | ***Lygocoris pabulinus – Liocoris tripustulatus*** | | ***Lygus punctatus – Liocoris tripustulatus*** | | ***Lygus punctatus – Lygocoris pabulinus*** | |
| --- | --- | --- | --- | --- | --- | --- | --- | --- |
|  | **PC1** | **PC2** | **PC1** | **PC2** | **PC1** | **PC2** | **PC1** | **PC2** |
| **bio01** | 0.2679286 | 0.2016702 | -0.2646626 | -0.2210181 | 0.26926845 | 0.2506863 | 0.2573119 | 0.0006114 |
| **bio02** | -0.2329192 | -0.0230372 | 0.21394043 | -0.0012749 | -0.2250619 | 0.0587691 | -0.242937 | 0.1260029 |
| **bio03** | 0.2307056 | 0.0709239 | -0.2433269 | -0.0690178 | 0.23169361 | 0.1196084 | 0.2129614 | -0.005001 |
| **bio04** | **-0.3083596** | -0.0638484 | **0.32065768** | 0.05154343 | **-0.2998357** | -0.0690462 | **-0.297678** | 0.0472449 |
| **bio05** | -0.0636349 | 0.2818849 | 0.04855481 | -0.2890833 | -0.0386429 | **0.3957113** | -0.134091 | 0.0576371 |
| **bio06** | **0.3035185** | 0.1447101 | **-0.3140669** | -0.1532796 | **0.29700206** | 0.1581214 | **0.2919485** | -0.053742 |
| **bio07** | **-0.3105028** | -0.0621705 | **0.32221278** | 0.04877496 | **-0.301811** | -0.0519992 | **-0.300653** | 0.0627043 |
| **bio08** | -0.1363585 | 0.0442105 | 0.14237324 | -0.0473345 | -0.1310581 | 0.0472575 | -0.149337 | 0.1533275 |
| **bio09** | 0.2751440 | 0.1956372 | -0.2762520 | -0.2026686 | 0.26599121 | 0.2340879 | 0.2817669 | -0.086887 |
| **bio10** | 0.0374842 | 0.2884480 | -0.0330844 | -0.2897209 | 0.06845455 | **0.4023628** | -0.016264 | 0.0706337 |
| **bio11** | 0.3005455 | 0.1433101 | -0.3104838 | -0.1533903 | 0.29431839 | 0.1699779 | 0.2885712 | -0.030757 |
| **bio12** | 0.2300942 | -0.333907 | -0.2136903 | 0.35164938 | 0.26091525 | -0.2491298 | 0.2211893 | 0.3412782 |
| **bio13** | 0.0919667 | **-0.3991558** | -0.0805657 | **0.37464092** | 0.1560101 | -0.2776939 | 0.0705578 | **0.5053567** |
| **bio14** | 0.2656448 | -0.1490478 | -0.2583032 | 0.18681588 | 0.25055841 | -0.2024213 | 0.2823996 | 0.0265405 |
| **bio15** | -0.2225025 | -0.1457526 | 0.20621115 | 0.12483395 | -0.2022734 | -0.0486807 | -0.236701 | 0.2408736 |
| **bio16** | 0.1170953 | **-0.4055945** | -0.1039726 | **0.38512511** | 0.17977472 | -0.2864871 | 0.0966059 | **0.4949831** |
| **bio17** | 0.2707439 | -0.1522297 | -0.2647313 | 0.1902812 | 0.25906365 | -0.193916 | 0.2831265 | 0.0364955 |
| **bio18** | 0.0385158 | **-0.4429416** | -0.0196265 | **0.4229269** | 0.03820487 | **-0.4203021** | 0.0593315 | **0.507483** |
| **bio19** | 0.2776043 | -0.0574578 | -0.2838679 | 0.07491494 | 0.27608967 | -0.0605039 | 0.2815585 | -0.016438 |
