# Supplementary material for: Climate change effect on the widely distributed Palearctic plant bug species (Insecta: Heteroptera: Miridae)
Source: PeerJ. 2024 Nov 22;12:e18377. doi: 10.7717/peerj.18377 (PMC11587874; doi:10.7717/peerj.18377)
Supplement: Supplemental Information 17 [file peerj-12-18377-s017.docx]

Table SI5**.** Log regression coefficients for the linear model analyses

| **Models** | ***Lygus punctatus + Lygocoris pabulinus*** | ***Liocoris tripustulatus + Lygocoris pabulinus*** | ***Liocoris tripustulatus + Lygus punctatus*** |
| --- | --- | --- | --- |
| **Classes** | *L. punctatus*: 0  *L. pabulinus*: 1 | *L. tripustulatus:* 0  *L. pabulinus:* 1 | *L. tripustulatus:* 0  *L. punctatus:* 1 |
| **Intercept** | 1.265610 | 0.049295 | -1.407235 |
| **bio01** | 0.755029 | – | – |
| **bio02** | -0.074651 | – | – |
| **bio08** | – | -0.321573 | -0.192497 |
| **bio09** | – | -0.869584 | -0.750891 |
| **bio11** | – | -0.224347 | -1.476057 |
| **bio13** | 0.583116 | 0.593941 | – |
| **bio17** | 0.725245 | 0.212391 | -0.308727 |
